# Total phenolic, flavonoid contents, *in-vitro* antioxidant activities and hepatoprotective effect of aqueous leaf extract of *Atalantia ceylanica*

**DOI:** 10.1186/1472-6882-14-395

**Published:** 2014-10-14

**Authors:** Chamira Dilanka Fernando, Preethi Soysa

**Affiliations:** Department of Biochemistry & Molecular Biology, Faculty of Medicine, University of Colombo, Kynsey Road, Colombo 08, Sri Lanka

**Keywords:** Phenolics, Flavonoids, *Atalantia ceylanica*, Aqueous extract, Antioxidants, Hepatoprotective

## Abstract

**Background:**

Decoction prepared from leaves of *Atalantia ceylanica* is used in traditional medicine in Sri Lanka for the treatment of various liver ailments since ancient times. Lyophilized powder of the water extract of *A. ceylanica* leaves was investigated for its phytochemical constituents, antioxidant and hepatoprotective activity *in-vitro*.

**Methods:**

The total phenolic and flavonoid contents were determined using Folin Ciocalteu method and aluminium chloride colorimetric assay respectively. The antioxidant activities of the decoction were investigated using 1,1-Diphenyl-2-picrylhydrazyl (DPPH), hydroxyl radical, nitric oxide scavenging assays and ferric ion reducing power assay. Hepatotoxicity was induced on porcine liver slices with ethanol to study hepatoprotective activity. Porcine liver slices were incubated at 37°C with different concentrations of the water extract of *A. ceylanica* in the presence of ethanol for 2 hours. The hepatoprotective effects were quantified by the leakage of alanine transaminase (ALT), aspartate transaminase (AST) and lactate dehydrogenase (LDH) to the medium. Thiobarbituric acid reactive substances (TBARS) assay was performed to examine the anti-lipid peroxidation activity caused by the plant extract.

**Results:**

The mean ± SD (n =9) for the levels of total phenolics and flavonoids were 4.87 ± 0.89 w/w% of gallic acid equivalents and 16.48 ± 0.63 w/w% of (-)-Epigallocatechin gallate equivalents respectively. The decoction demonstrated high antioxidant activity. The mean ± SD values of EC_50_ were 131.2 ± 36.1, 48.4 ± 12.1, 263.5 ± 28.3 and 87.70 ± 6.06 μg/ml for DPPH, hydroxyl radical, nitric oxide scavenging assays and ferric ion reducing power assay respectively.

A significant decrease (*p* <0.05) was observed in ALT, AST and LDH release from porcine liver slices treated with *A. ceylanica* extract at a concentration of 2 mg/ml in the presence of ethanol (5 M) compared to that of ethanol (5 M) treated slices. Furthermore, a reduction in lipid peroxidation was also observed in liver slices treated with the leaf extract of *A. ceylanica* (2 mg/ml) compared to that of ethanol induced liver toxicity (*p* <0.05).

**Conclusions:**

The results suggest that aqueous extract of *A. ceylanica* exerts hepatoprotective activity against ethanol induced liver toxicity of porcine liver slices which can be attributed to the antioxidant properties possessed by the plant material.

**Electronic supplementary material:**

The online version of this article (doi:10.1186/1472-6882-14-395) contains supplementary material, which is available to authorized users.

## Background

During hepatic drug biotransformation, free radicals are continuously generated. Free radicals are highly reactive, unstable molecules that react rapidly with adjacent molecules via a variety of reactions including: hydrogen abstraction, electron capturing and electron sharing leading to lipid peroxidation, protein oxidation, DNA strand breaks, and modulation of gene expression [1]. Experimental evidence shows that these free radicals are involved in liver diseases and also lead to atherosclerosis, cancer, stroke, asthma, arthritis and other age related diseases [2, 3].

Plants as natural source of antioxidants have the potential to scavenge free radicals and inhibit their generation. It is a well established fact that the mechanism of hepatoprotective effects of certain drugs are related to their antioxidant capacity in scavenging free radicals and reactive oxygen species [4]. Since, toxic effects of synthetic antioxidants have been reported, the interest for searching of natural antioxidants of plant origin has increased [5]. Use of plant derived drugs in medical practice has shown that they are relatively non-toxic, safe and even free from serious side effects [6].

*Atalantia ceylanica* (Family: Rutaceae) locally known as “Yakinaran” is a dicotyledonous densely branched shrub up to 2.5 m height distributed in Sri Lanka and Southern India [7]. The juice of the leaves is used in the preparation of pills administered for catarrh, bronchitis and other chest complaints [8]. The root is used in the treatment of ague [8]. Decoction prepared from leaves of *Atalantia ceylanica* is used in the treatment of liver diseases by traditional medical practitioners of Sri Lanka.

Due to the lack of scientific investigations carried out so far, the current research was launched to determine the phytochemical composition, antioxidant and hepatoprotective activity of the decoction prepared from *Atalantia ceylanica*.

## Methods

### Chemicals and equipment

The chemicals gallic acid, Folin Ciocalteu reagent, trichloroacetic acid, 2-deoxy-D-ribose and ethylenediamine tetra acetic acid (EDTA) were purchased from Sigma Chemicals Co. (P.O. Box 14508, St. Louis, MO 63178 USA). 1,1-Diphenyl-2-picrylhydrazyl (DPPH) free radical, (-)-epigallocatechin gallate, aluminium chloride and sulfanilamide were purchased by Fluka (Fluka chemie GmbH, CH-9471 Buchs). L-ascorbic acid, hydrogen peroxide, N-(1-naphthyl)-ethylene diamine dihydrochloride and ethanol were purchased from BDH Chemicals (BDH Chemicals Ltd, Poole, England). Sodium nitroprusside was purchased from Qualigens (A division of GlaxoSmith Kline Pharmaceuticals Ltd). Ferric chloride, potassium ferricyanide and sodium nitrite were purchased from Riedel De Haen Ag, Wunstorfer Strasse 40, SEELZE1, D3016, Germany.

Lactate dehydrogenase (LDH) enzyme assay kit was purchased from DiaSys (Alte Strasse 9, 65558, Holzheim, Germany). Alanine transaminase (ALT) and Aspartate transaminase (AST) enzyme assay kits were purchased from POINTE, SCIENTIFIC, INC (5449 Research Drive, Canton MI 48188, USA).

SHIMADZU UV 1601 UV Visible spectrophotometer (Shimadzu Corporation, Kyoto, Japan) was used to read the absorbance. Deionized water was obtained from LABCONCO WATER PRO-PS UV ultra filtered water system (LABCONCO Corporation, Kansas city, Missouri).

### Plant materials

The leaves of *Atalantia ceylanica* (Yakinaran) were collected from Nachchaduwa, Anuradhapura, Sri Lanka and was identified and confirmed by Department of Botany, Bandaranaike Memorial Ayurvedic Research Institute, Nawinna, Colombo, Sri Lanka. Voucher specimens are deposited at the above premises.

### Preparation of the decoction

Washed plant leaves were dried until a constant weight was achieved. Dry weight of 30 g of plant material (n =3) was ground to a fine powder and boiled with 800 ml of deionized water until total volume reduced to 100 ml (1/8th of the original volume). The decoction was sonicated and filtered. The filtrate was centrifuged (2000 rpm, 10 min) and the supernatant was freeze dried. The freeze dried samples were weighed, and stored at -20°C in sterile tubes until further use. Lyophilized samples of *A. ceylanica* were prepared in triplicates and the yield was calculated as a percentage of dry weight.

### Determination of total phenolic content

Total phenolic content of decoctions of *A. ceylanica* was determined by Folin Ciocalteu method [9]. Calibration curve was constructed using gallic acid standards and the total phenolic content was expressed as w/w% gallic acid equivalents.

### Determination of flavonoid content

The flavonoid content was measured by the aluminium chloride colorimetric assay [10]. Calibration curve was plotted using (-)-epigallocatechin gallate (EGCG) standards and flavonoid content was expressed as w/w% EGCG equivalents.

### 1,1-Diphenyl-2-picrylhydrazyl (DPPH) free radical scavenging activity

Free radical scavenging ability of the decoction prepared was assessed by DPPH radical scavenging method with slight modification [11]. DPPH reagent prepared in absolute ethanol (100 μM, 750 μl) was added to test sample (250 μl) and the mixture was allowed to stand for 30 minutes in the dark. Absorbance was measured at 517 nm. Percentage inhibition was calculated according to equation 1:
1

L-Ascorbic acid was used as the reference standard antioxidant. The effective concentration needed to scavenge DPPH free radical by 50% (EC_50_) was calculated by regression analysis of the dose response curve plotted between percentage inhibition versus concentration of the test samples and the standard.

### Hydroxyl radical (HO^.^) scavenging activity

Hydroxyl radical scavenging activity was measured based on the competition between deoxyribose and the test compound (the plant extract) to react with hydroxyl radical generated from Fe^2+^/Ascorbate/EDTA/H_2_O_2_ system according to the procedure as previously described with slight modification [12]. Gallic acid was used as the reference standard antioxidant. The percentage scavenging of hydroxyl radical for *A. ceylanica* and the standard antioxidant was calculated according to equation 1. EC_50_ was calculated as described previously.

### Nitric oxide radical (NO^.^) scavenging activity

NO^.^ scavenging activity of the decoctions prepared was measured based on Griess - Ilosvay reaction with slight modification [13]. The interference from the plant extract with the pink chromophore formed was minimized by background subtraction of absorbance for respective concentrations. L-Ascorbic acid was used as the reference standard antioxidant. The percentage scavenging of NO^.^ for *A. ceylanica* and the standard antioxidant was calculated according to equation 1. EC_50_ was calculated as described previously.

### Ferric ion reducing power assay

The ferric ion reducing power of the decoctions prepared was determined according to a method described previously [14]. L-ascorbic acid was used as the reference standard antioxidant. Dose response curve was plotted between the absorbance versus concentrations of plant extracts or standards by linear regression analysis. EC_50_ was defined as the corresponding concentration of the plant extract which gives an absorbance value of 0.5.

### Porcine liver tissue collection

Porcine liver tissue of either sex was obtained from the registered slaughter house in Dematagoda, Sri Lanka (See Additional file 1) with permission obtained from the chief municipal veterinary surgeon. A sample of liver tissue without distinction of lobes was excised using sterile scalpel blades and transferred immediately into ice cold sterile Krebs Ringers-4-(2-hydroxyethyl)-1-piperazineethanesulfonic acid (HEPES) buffer (KRHB) and transported to the laboratory on the day of the experiment within 5 minutes in a ice bath.

### Preparation of medium

Tissue slices were prepared in sterile KRHB and maintained according to a method described previously [15, 16]. The buffer (KRHB) composition includes HEPES (2.5 mM), NaCl (118 mM), KCl (2.85 mM), CaCl_2_ (2.5 mM), KH_2_PO_4_ (1.5 mM), MgSO_4_ (1.18 mM) and glucose (4.0 mM). The pH was adjusted to 7.4 by 1 N NaOH. The medium was autoclaved for sterilization.

### Hepatoprotective activity of *A. ceylanica*decoction *in vitro*

Porcine liver tissue was washed several times in fresh ice cold KRHB and cut into thin slices (20–25 mg) using sharp sterile scalpel blades. Liver slices (100 mg) were transferred into clean sample collection glass vials filled with KRHB (3.0 ml). Vials were capped and pre-incubated for 30 minutes in a shaker water bath at 37°C. Liver slices (100 mg) were drained carefully and transferred into separate vials having different concentrations of *A. ceylanica* (400, 1000 and 2000 μg/ml) co-exposed with ethanol (5 M) and incubated at 37°C for 2 hours. The final volume was adjusted to 3.0 ml with KRHB. Same experiment was carried out with the plant extract in the absence of ethanol to assess any hepatotoxic effect induced by the plant extract itself. A control was carried out in KRHB (3.0 ml) and ethanol (5 M) was used to induce hepatotoxicity. After the incubation period, the spent media were collected and the liver slices were homogenized at 4°C, the homogenates were sonicated for 4 seconds and centrifuged at 4°C. The supernatants of the tissue homogenates and the media obtained from post incubation of tissues were assayed for alanine transaminase (ALT), aspartate transaminase (AST) and lactate dehydrogenase (LDH).The percentage cytotoxicity was calculated using equation 2:
2

Where Total Enzyme activity = Enzyme activity in the medium + Enzyme activity in the tissue homogenate, Medium = Medium used for the incubation of liver tissue

The tissue homogenates were also assayed for total protein content and lipid peroxides formed [17, 18]. Standard curves were plotted using bovine serum albumin (BSA) and 1,1,3,3-Tetraethoxypropane (TEP) standards respectively and amount of lipid peroxides formed was expressed as micrograms of malondialdehyde (MDA) equivalents formed per gram of protein.

### Statistical analysis

A minimum of three independent experiments were carried in triplicates unless otherwise specified. Students T test was performed for statistical analysis and results are presented as mean ± standard deviation (Mean ± SD). Value of *p* <0.05 was considered as significant. Regression analysis and statistical analysis were carried out using Microsoft Excel. Calibration curves of the standards were considered as linear if R^2^ > 0.99. EC_50_ values were calculated from either linear or logarithmic dose response curves where R^2^ > 0.90.

## Results and discussion

### Extraction yield, phenolic and flavonoid contents

The extraction yield obtained for lyophilized samples of *A.ceylanica* was 8.56 ± 1.50% of dry weight. Phenolic compounds contain hydroxyl groups which contribute to the free radical scavenging and act as primary antioxidants [19]. *A. ceylanica* exhibited total phenolic content of 4.87 ± 0.89 w/w% gallic acid equivalents (Table 1). In a similar study carried out using the ethanolic leaf extract of *Atalantia monophylla* (Family: Rutaceae), has shown that the total phenolic content present was 560 mg/g (56% w/w% gallic acid equivalents) which is very high compared to the present study [20]. Higher solubility of phenolic compounds in ethanol compared to water may be associated with this observation, apart from the duration of extraction, temperature used and method of extraction [21].

**Table 1:** **The extraction yield, total phenolic content and flavonoid content obtained for** ***A. ceylanica***

**Extraction yield (w/w% of dry weight)**	**8.56 ± 1.50 (n =3)**
**Phenolic content (w/w% Gallic acid equivalents)**	**4.87 ± 0.89 ( n =9)**
**Flavonoid content (w/w% (-)-Epigallocatechin gallate equivalents)**	**16.48 ± 0.63 (n =9)**

Flavonoids are considered as potential antioxidants exerting their antioxidant activity by the mechanisms of radical scavenging and metal ion chelation to inhibit lipid peroxidation [22]. Aluminium chloride colorimetric assay yielded total flavonoid content of 16.48 ± 0.63 w/w% (-)-Epigallocatechin gallate (EGCG) equivalents for *A. ceylanica* (Table 1). Methanolic extract *of Aegle marmelos* leaves which belongs to the same family of *A.ceylanica* exhibits a total phenolic content of 9.84 ± 0.023 mg/kg gallic acid equivalents and a total flavonoid content of 8.248 ± 0.029 mg/kg EGCG equivalents [23]. This indicates that the aqueous extract of *A. ceylanica* possess a higher content of phytochemicals compared to *Aegle marmelos*.

### DPPH radical scavenging activity

**Figure 1:**
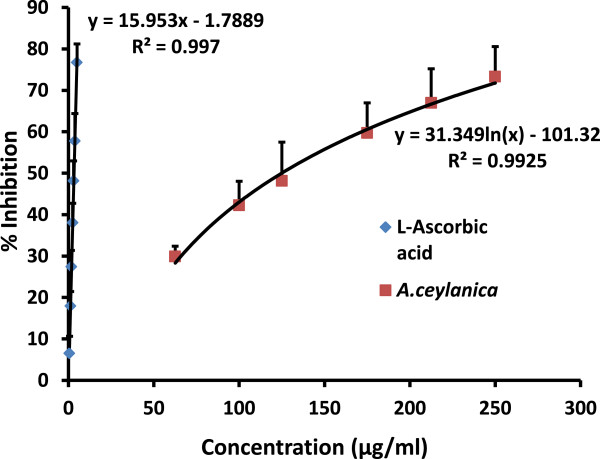
**The dose response curves for percentage scavenging of DPPH by** ***A. ceylanica*** **decoction in comparison with L-Ascorbic acid.** The results are presented as mean + SD for *A. ceylanica* (n =9) and L-Ascorbic acid (n =9).

1,1-Diphenyl-2-picrylhydrazyl (DPPH) free radical was used to determine hydrogen donating ability of the plant extracts. DPPH reacts with hydrogen donors (free radical scavengers) to yield a stable product 1,1-Diphenyl-2-picrylhydrazine resulting in a color change from purple to yellow [24]. In the current study, the values for EC_50_ obtained for DPPH assay were 131.2 ± 36.1 μg/ml and 3.30 ± 0.27 μg/ml for *A. ceylanica* and L-Ascorbic acid respectively (Figure 1). The results indicate that hydrogen donating ability of L-Ascorbic acid is greater than *A. ceylanica*. A previous study carried out with *Murraya koenigii* (Indian curry leaf tree) which belongs to the same family, Rutaceae has shown that the EC_50_ values for DPPH scavenging were 187 μg/ml and 917 μg/ml for its methanolic and aqueous leaf extracts respectively [25]. These values indicate that decoction prepared from *A. ceylanica* leaves is a better scavenger of free radicals than *Murraya koenigii* aqueous leaf extract and the EC_50_ value is comparable to the EC_50_ value of the methanolic leaf extract.

### Hydroxyl radical (HO^.^) scavenging activity

**Figure 2:**
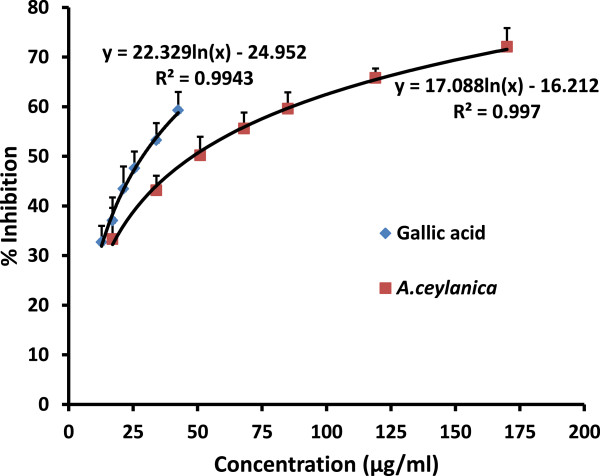
**The dose response curves for percentage scavenging of hydroxyl radicals by** ***A.ceylanica*** **decoction and Gallic acid.** The results are presented as mean + SD for *A. ceylanica* (n =9) and Gallic acid (n =9).

Hydroxyl radical is an extremely reactive free radical formed in biological systems which can react and damage biomolecules found in living cells. Hydroxyl radicals can initiate lipid peroxidation of the cell membrane. Iron catalysed ^.^OH radical formation degrades deoxyribose to thiobarbituric acid reactive substances (TBARS) which generates a pink chromogen on heating with thiobarbituric acid (TBA) [12]. In the present investigation, *A. ceylanica* demonstrated an EC_50_ value of 48.4 ± 12.1 μg/ml compared to gallic acid which showed a value of EC_50_ = 27.69 ± 4.14 μg/ml. The results obtained strongly indicate that the aqueous extract of *A. ceylanica* leaf is capable of protecting deoxyribose in the presence of hydroxyl radicals in a dose dependent manner (Figure 2). Ethanolic extract of *Ruta graveolens* leaf and hydro-ethanolic extract of *Citrus aurantium* leaf, the plants which belong to the same family have shown an EC_50_ value of 160.09 μg/ml and 96.76 ± 0.95 μg/ml respectively indicating a higher capacity to scavenge hydroxyl radical by *A. ceylanica* [26, 27].

### Nitric oxide radical (NO^.^) scavenging activity

**Figure 3:**
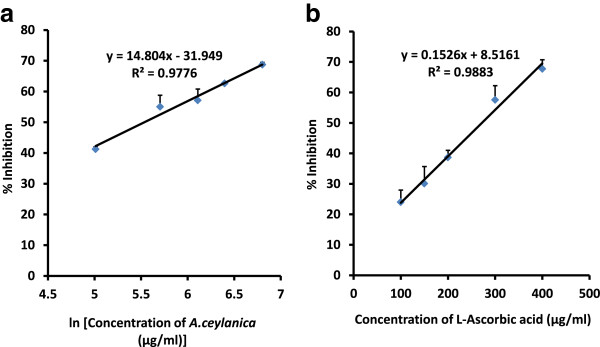
**The dose response curve for percentage inhibition of nitric oxide radicals by** ***A. ceylanica*** **(a) and L-Ascorbic acid (b).** A linear relationship was observed between % I versus natural logarithm of concentration of *A. ceylanica*. The results are presented as mean + SD of three independent experiments.

Formation of NO^.^ is elevated in infections and inflammation, and in the aerobic environment NO^.^ reacts with oxygen to produce strong oxidants such as peroxynitrite anion (ONOO^-^). Although NO^.^ does not interact with biological macromolecules directly, peroxynitrite anion (ONOO^-^) will give rise to adverse effects such as DNA fragmentation, cell damage and neuronal cell death [28]. Nitric oxide generated spontaneously from sodium nitroprusside (SNP) in aqueous solution at physiological pH interacts with oxygen to produce nitrite ions causing diazotization of sulphanilamide which in turn undergoes coupling with naphthylethylenediamine dichloride forming an azo-dye. Scavengers of nitric oxide compete with oxygen which leads to reduced production of nitrite ions. The EC_50_ values obtained were 263.5 ± 28.3 and 276.3 ± 25.8 μg/ml for *A. ceylanica* and L-ascorbic acid respectively (Figure 3). These results suggest that there is no significant difference between NO scavenging abilities of *A. ceylanica* and L-Ascorbic acid (*p* >0.05). *Citrus aurantium* and *Citrus acidissima* are two plants which belong to the family of Rutaceae. *A. ceylanica* leaf has a higher antioxidant potential to scavenge NO radicals compared to hydro-ethanolic extract of *Citrus aurantium* leaf (EC_50_ = 765.41 ± 0.82 μg/ml) but a lesser scavenging ability compared to methanolic extract of *Citrus acidissima* leaf (EC_50_ = 152 μg/ml) [27, 29].

### Ferric ion reducing power assay

**Figure 4:**
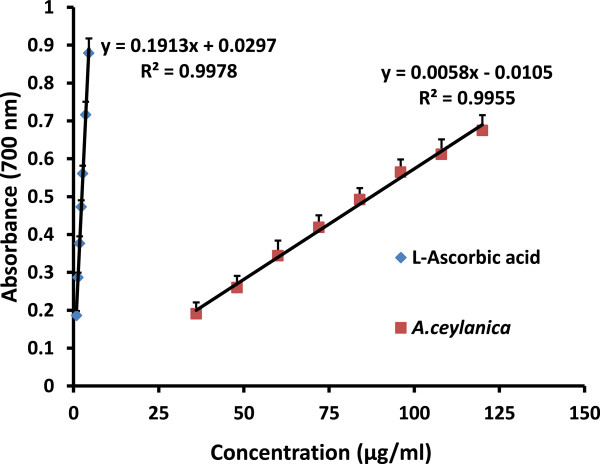
**The reduction capability of different concentrations of** ***A. ceylanica*** **decoction in comparison with L-Ascorbic acid.** The results are presented as mean + SD for *A. ceylanica* (n =9) and L-Ascorbic acid (n =9).

Ferric ion reducing power assay measures the electron-donating capacity of an antioxidant [30]. The presence of reducing agents (i.e. antioxidants) causes the reduction of the Fe^3+^/ferricyanide complex to the ferrous form. The absorbance measured at 700 nm of the resultant blue-green coloured solution is proportional to the amount of Fe^2+^ in the system. Therefore an increased absorbance is indicative of higher reducing power. The reducing power of *A. ceylanica* leaf extract as well as the standard L-Ascorbic acid increased progressively in a dose dependant manner over the concentration ranges studied, however the reducing power of L-Ascorbic acid was greater than *A.ceylanica*. The corresponding concentrations of L-Ascorbic acid and *A. ceylanica* to produce a blue-green colored product of 0.5 absorbance (EC_50_) were 2.78 ± 0.30 and 87.70 ± 6.06 μg/ml respectively. The reduction capability of *A. ceylanica* aqueous leaf extract compared to L-ascorbic acid is presented in Figure 4. The results obtained for antioxidant activity experiments are tabulated in Table 2.

**Table 2:** **The EC**_**50**_ **values for antioxidant tests for aqueous leaf extract of** ***A. ceylanica*** **with respect to reference standard antioxidants**

Antioxidant test	***A. ceylanica***(μg/ml)	Ascorbic acid (μg/ml)	Gallic acid (μg/ml)
**DPPH assay (n =9)**	**131.2 ± 36.1**	**3.30 ± 0.27**	**-**
**HO** ^**.**^ **scavenging (n =9)**	**48.4 ± 12.1**	**-**	**27.69 ± 4.14**
**NO** ^**.**^ **scavenging (n =3)**	**263.5 ± 28.3**	**276.3 ± 25.8**	**-**
**Fe** ^**3+**^ **reducing power (n =9)**	**87.70 ± 6.06**	**2.78 ± 0.30**	**-**

### Hepatoprotective activity of *A. ceylanica*aqueous extract

Liver slices have been successfully used for assaying hepatoprotective activity of compounds such as curcumin [31] and *Pterocarpus marsupium* extract [32]. Further it has been found that, liver tissue collected from slaughter house is a useful model to study hepatotoxicity of different substances on the organ level [33]. There is a similarity in liver specific metabolic activities with porcine liver cells with that of human liver cells [34]. The present study was therefore carried out using liver slices prepared from fresh porcine liver obtained from the slaughter house.

**Figure 5:**
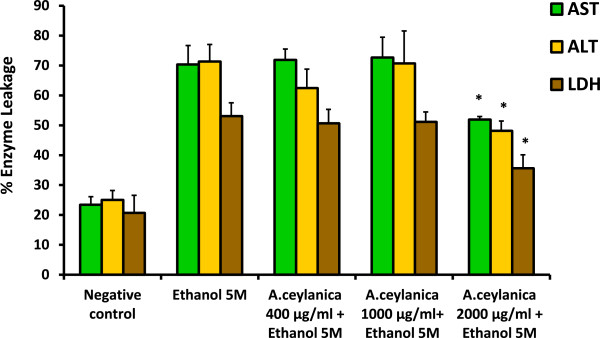
**Percentage enzyme leakage of porcine liver slices after 2 hour exposure to medium (negative control), ethanol (5 M) and different concentrations of** ***A. ceylanica*** **(400, 1000, 2000 μg/ml) with ethanol (5 M).** Each value represents mean + SD (n =3). ^*^ *p* <0.05 when compared to ethanol (5 M) treated sample for the corresponding enzyme assayed. AST = Aspartate transaminase, ALT = Alanine transaminase, LDH = Lactate dehydrogenase.

Trial experiments were carried out to optimize the conditions to cause liver damage by ethanol to investigate the protective effect of *A. ceylanica* aqueous extract on liver slices. It was found that the concentration of the ethanol and exposure time required to induce liver damage was 5 M and 2 hours respectively. The activities of the three enzymes ALT, AST and LDH were increased in the porcine liver slices treated with ethanol (5 M) indicating cellular leakage and loss of functional integrity of the cell membrane [35]. Percentage AST, ALT, and LDH released in liver slices treated with ethanol for 2 hours were 70.36%, 71.33% and 53.06% respectively and for untreated liver slices (control), the values were 25.05%, 23.43% and 20.67% respectively (Figure 5). There was a significant (*p* <0.05) reduction of percentage release of ALT, AST and LDH after treatment with *A. ceylanica* at a concentration of 2 mg/ml (Figure 5)*.* This confirms the protective effect of the decoction prepared from *A. ceylanica* against ethanol induced hepatic damage. The prevention of the leakage of intracellular enzymes may be attributed to the membrane stabilizing activity caused by the phytochemicals present in the plant extract.

**Figure 6:**
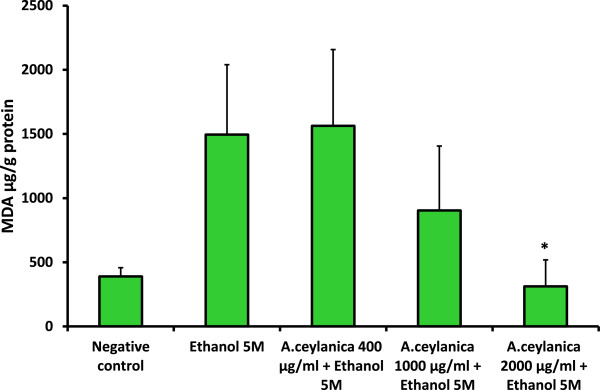
**MDA equivalents formed in liver tissue homogenates after exposure to different conditions for 2 hour incubation period.** Each value represents mean + SD (n =3). ^*^ *p* <0.05 when compared to ethanol (5 M) treated sample. MDA = Malondialdehyde.

In the pathogenesis of ethanol-induced liver injury, oxidative stress plays an important role [36]. Free radicals generated on ethanol consumption react with various cellular components and cause damage to the tissues eventually forming products such as lipoperoxides, conjugated dienes and malondialdehyde (MDA) with simultaneous reduction of levels of antioxidants like Vitamin E and glutathione in the tissues [37]. The porcine liver slices being treated with ethanol (5 M) produced high amount of lipid peroxides (1495.5 ± 544.8 μg/g amount of MDA equivalents) compared to untreated liver slices, indicating the oxidative damage caused by exposure to high levels of ethanol (Figure 6). Treatment of porcine liver slices with ethanol (5 M) with 2 mg/ml of plant extract for 2 hours has reduced the formation of lipid peroxides significantly (*p* <0.05) compared to the sample treated with 5 M ethanol alone (Figure 6). The results indicate the contribution of the secondary metabolites of *A. ceylanica* to reduce or scavenge lipid peroxides generated from free radicals. The plant extracts alone in KRHB did not show any toxicity on liver tissue over the concentrations studied. It is well documented that antioxidants protect against toxicity caused by reactive oxygen species (ROS) in the prevention of their generation, disruption of attack, scavenging of reactive metabolites and enhancing the resistance to ROS attack on sensitive biological targets [38].

A similar study carried out by Sinha *et al*. [39], on hepatoprotective activity of *Picrorhiza kurroa* Royle Ex. Benth aqueous root extract (Family: Scrophulariaceae) using mouse liver slice culture, the authors concluded that the high antioxidant activity of this plant is responsible for the suppression of alcohol induced toxicity. In a recent study carried out by Shokrzadeh and coauthors (2014), have found that ethanolic extract of *Zataria multiflora* can exert hepatoprotective activity against liver toxicity induced by cyclophosphamide (CP) in mice [40]. Although CP is extensively used in chemotherapy, it possesses a wide spectrum of side effects including hepatotoxicity. Based on their results the authors concluded that *Zataria multiflora*, could be used concomitantly as a supplement agent against hepatotoxicity for the patients undergoing chemotherapy with CP.

## Conclusion

The results obtained in this study indicate that the decoction prepared from *A. ceylanica* leaves possess effective hepatoprotective activity against ethanol induced toxicity in porcine liver slices. This can be attributed to the radical scavenging capacity of the plant extract hence justifying the use of this plant material in the treatment of various liver diseases in traditional medicine. The pharmacological profiles of this plant extract based on *in vivo* studies and clinical trials should be further investigated.

## Electronic supplementary material

Additional file 1: **IACUC Principles and procedures of animal care and use.** (PDF 271 KB)
