# Supplementary material for: Total phenolic, flavonoid contents, in-vitro antioxidant activities and hepatoprotective effect of aqueous leaf extract of Atalantia ceylanica
Source: BMC Complement Altern Med. 2014 Oct 14;14:395. doi: 10.1186/1472-6882-14-395 (PMC4210483; doi:10.1186/1472-6882-14-395)
Supplement: Supplementary file 1 — Additional file 1: IACUC Principles and procedures of animal care and use. (PDF 271 KB) [file 12906_2014_1975_MOESM1_ESM.pdf]

IACUC PRINCIPLES AND PROCEDURES OF ANIMAL CARE AND USE

Institutional Animal Care & Use Committee,
Division of Comparative Medicine & Division of Research Integrity & Compliance,
Office of Research, University of South Florida
OLAW PHS # A-4100-01; USDA # 58-R-0015; AAALAC # 000434

**I.**	**University Principles and Procedures Regarding Use of Animals in Research, Teaching, or Testing..**	**1**
**II.**	**Institutional Official…………………………………………………………………………………..**	**2**
**III.**	**Institutional Animal Care and Use Committee………………………………………………………..**	**2**
**IV.**	**Division of Research Integrity & Compliance and Research Grants Regarding Animal Care and Use**	**7**
**V.**	**Division of Comparative Medicine…………………………………………………………..................**	**8**
**VI.**	**Preclinical GLP Studies………………………………………………………………………………...**	**9**
**VII.**	**Occupational Health and Safety……………………………………………………........................**	**10**
**VIII.**	**Certifying Adequacy of Personnel Training and Experience……………………………......................**	**10**
**IX.**	**Reporting Deficiencies in Animal Care or Treatment…………………………………………………..**	**12**
**X.**	**Suspension and Appeal Process of Animal Use Privileges……………………………………………..**	**13**
**XI.**	**Transportation, Relocation or Reassignment of Animals………………………………………………..**	**14**
**XII.**	**Avoidance and Alleviation of Research Animal Pain and Discomfort……………………………………**	**15**
**XIII.**	**Anesthesia and Analgesia………………………………………………………………………………..**	**17**
**XIV.**	**Controlled Substances…………………………………………………………………………………….**	**17**
**XV.**	**Surgical Techniques and Post-operative Care…………………………………………………………..**	**18**
**XVI.**	**Multiple Major Survival Surgical Procedures…………………………………………………………….**	**20**
**XVII.**	**Antiserum Production……………………………………………………………………………………..**	**20**
**XVIII.**	**Monoclonal Antibody Ascites Production……………………………………………………………….**	**22**
**XIX.**	**Animal Identification and Animal Medical Records……………………………………………………..**	**22**
**XX.**	**Animal Euthanasia…………………………………………………………………………......................**	**24**
**XXI.**	**Animal Acclimation and Conditioning…………………………………………………………………….**	**25**
**XXII.**	**Animal Facilities……………………………………………………………………………………………**	**25**
**XXIII.**	**Studies of Wild Animals In or From Natural Settings……………………………………………………..**	**26**
**XXIV.**	**Conflict of Interest………………………………………………………………………………………….**	**29**
**XXV.**	**Public Records……………………………………………………………………………………………..**	**29**

I. University Principles and Procedures Regarding Use of Animals in Research, Teaching, or Testing

I.1. The University of South Florida (University) affirms that respect for all forms of life is an inherent characteristic of biological and medical scientists who conduct research involving animals, that the respectful treatment, care and use of animals involved in research is an ethical and scientific necessity, and that the use of animals in research and teaching contributes to the advancement of knowledge and the acquisition of understanding.

I.2. The University has established and provides resources for an Animal Care and Use Program that is managed in accordance with the *Guide for the Care and Use of Laboratory Animals* (*Guide*) (viewable at http://www.nap.edu/catalog.php?record_id=12910), the *Animal Welfare Act,* (viewable at http://www.aphis.usda.gov/animal_welfare/awa_info.shtml), *Title 9 Code of Federal Regulations Subchapter A, "Animal Welfare", Parts 1-3* (AWA), (viewable at http://www.a phis.usda.gov/animal_welfare/downloads/awr/awr.pdf), the *Public Health Service Policy on Humane Care and Use of Laboratory Animals* (*PHS Policy*), (viewable at http://grants.nih.gov/grants/olaw/references/phspol.htm), University Policy #0-308, *Use of Animals in Research, Testing, and Teaching,* (viewable at http://generalcounsel.usf.edu/policies-and-procedures/pdfs/policy-0-308.pdf) and the Institutional Animal Care and Use Committee's *IACUC Principles and Procedures of Animal Care and Use* (*IACUC Principles and Procedures*) (viewable at http://www3.research.usf.edu/cm/docs/cmdc/IACUCPolicies.pdf . These written IACUC Principles and Procedures and all associated relevant documents are reviewed and updated semi-annually by a quorum of the regular members of the IACUC. The University program and facilities for animal care and use are fully accredited by the *Association for Assessment and Accreditation of Laboratory Animal Care International* (AAALAC) accessible at http://www.aaalac.org/ .

I.3. Preclinical studies that support or are intended to support applications for research or marketing permits for products regulated by the Food and Drug Administration should be conducted in accordance with *Title 21 Code of Federal Regulations Part 58 Good Laboratory Practice for Nonclinical Laboratory Studies* Viewable at http://www.access.gpo.gov/nara/cfr/waisidx_99/21cfr58_99.html .

I.4. Studies of wild animals in or derived from natural settings are conducted in accordance with the *Guidelines of the American Society of Mammalogists for the Use of Wild Mammals in Research*, viewable at http://www3.research.usf.edu/cm/docs/ASM-Guidelines_2011.pdf , the *Guidelines for Use of Fishes in Field Research,* viewable at http://www.nal.usda.gov/awic/pubs/Fishwelfare/ASIH.pdf, the *Guidelines for Use of Live Amphibians and Reptiles in Field and Laboratory Research* at http://www3.research.usf.edu/cm/docs/ASIH_HACC_Amphib_Reptile_Guide.pdf , and *Guidelines to the Use of Wild Birds in Research* at http://www3.research.usf.edu/cm/docs/Wild_Bird_Guidelines_2010.pdf .

II. Institutional Official

II.1. The Institutional Official is appointed by the University President and is the administrative official responsible for the Animal Care and Use Program. Overall program direction is a shared responsibility of the Institutional Official, Directing Veterinarian, and Institutional Animal Care and Use Committee (IACUC). The Institutional Official has appointed an IACUC, including alternates, which is responsible for oversight and evaluation of the animal care and use program, its procedures and facilities to ensure that they are consistent with the recommendations of the Guide, AWA, PHS Policy, University Policy #0-308, and the IACUC Principles and Procedures, and are fully accredited by AAALAC.

III. Institutional Animal Care and Use Committee

III.1. New IACUC members are required to complete the two web-based training modules viewable at http://www.aalaslearninglibrary.org/ entitled “Essentials for IACUC Members” and “Working with the IACUC”. IACUC members with VA affiliation must also complete “Working with the VA IACUC.” New IACUC members are required to attend a formal session of orientation and training by the IACUC administrative staff regarding the functions of the IACUC, and the regulations, principles and procedures that govern the University’s Animal Care and Use Program, as itemized in PHS Policy IV.B.1-8. IACUC members or alternates that do not comply with training requirements are provided written notification from the IACUC Chairperson that training requirements must be completed within 30 days. Failure to comply after said written notification can result in dismissal from the IACUC by the Institutional Official.

III.2. The IACUC has authority to review, approve, or require modification in order to secure approval, or disapprove proposed research or teaching using vertebrate animals, review the facilities and program for animal care and use, including laboratories outside of animal facilities where procedures are performed, review and, if warranted, investigate concerns involving the care and use of animals, prepare written reports of its evaluations, make recommendations to the Institutional Official concerning any aspect of the animal care and use program, and suspend any activity involving animals that does not conform to the Guide, AWA, PHS Policy or the IACUC Principles and Procedures.

III.3. The IACUC consists of not less than five members, each of whom fulfill one or more membership category(s), either (1) a Doctor of Veterinary Medicine with training or experience in laboratory animal science and medicine who has direct or delegated program responsibility for activities involving animals at the institution, (2) a practicing scientist experienced in research involving animals, (3) a member who is not affiliated with the University other than as a member of the IACUC and is not a member of the immediate family of a person who is affiliated with University, and (4) a member whose primary concerns are in a nonscientific area and who represents general community interests. IACUC membership includes at least one representative from the College of Health Sciences, the College of Arts and Sciences, the James A. Haley Veteran’s Administration Hospital, and the H. Lee Moffitt Cancer Research Center. IACUC membership includes a biologist who can provide the IACUC with an understanding of the nature and impact of proposed field investigations, the housing and care of the species to be studied, and the risks associated with maintaining wild vertebrates in captivity.

III.4. IACUC members and alternates are appointed by the Institutional Official, and are listed on the IACUC rosters submitted to the PHS. Multiple alternates are appointed to fulfill one or more of the four specific membership categories mentioned above. An alternate may serve for any regular member provided the alternate fulfills the specific membership requirement(s) of the regular member for whom he/she is substituting. IACUC members must attend each regular monthly meeting and must notify the IACUC administrative staff of planned absences prior to protocol review assignments. When an IACUC member is unable to attend a meeting after protocol review assignments have been made, the IACUC member must make electronic review entries directly on the electronic IACUC (e-IACUC) application using the Applications for Research Compliance (ARC) system for each assigned protocol prior to the monthly meeting. When a regular member is

unavailable to serve, the IACUC administrative staff identify an alternate that fulfills the same membership requirement(s) of the absent regular member and arranges for their participation to ensure that the committee is properly constituted at all times. Although members and alternates may not contribute to a quorum or act in an official IACUC capacity at the same time, alternates are encouraged to attend all IACUC meetings. Alternates receive the same training and orientation as IACUC members, and are expected to "vote their conscience" as opposed to representing the position of the regular member. The Institutional Official can dismiss IACUC members or alternates absent from more than three regular (or assigned) monthly meetings in a calendar year.

III.5. All live vertebrate animal use, including field studies, conducted by University faculty, students, or staff, or supported by University funds, must first be described in a draft e-IACUC application using the ARC system and be pre-reviewed by University veterinarians prior to its submission to the IACUC for full committee consideration. All vertebrate animal use must then be proposed to, and approved by the IACUC as an e-IACUC protocol prior to the initiation of that activity, regardless of where it will be performed.

III.6. Principal Investigators (PI) developing applications for VA funded Merit Review grants, which are reviewed and approved by the VA Central Office, must submit the animal component for their research project to the IACUC using the VA Animal Component entitled "*Animal Component of Research Proposal*" (ACORP) viewable at http://www.research.va.gov/programs/animal_research/. VA R&D review must be secured prior to IACUC review. All ACORP applications to the IACUC will be assigned a primary reviewer that is a VA resident scientist or administrator familiar with the ACORP format and a regular member or alternate member of the IACUC. PIs developing applications for the Department of Defense may submit applications for review using the *Department of Defense Animal Use Protocol.*

III.7. PIs that intend to create, or have others create mice locally by pronuclear microinjection of DNA, or by blastocyst microinjection of embryonic stem cells that have been electroporated with DNA, or by other methods of genetic engineering involving recombinant DNA, must first secure IACUC approval, which describes the genotype or line of mice to be created, and is contingent on approval of a *Non-Exempt Recombinant DNA Registration* application by the Institutional Biosafety Committee. All colonies of locally produced or maintained mice must be represented by an approved e-IACUC murine colony only protocol. Murine colony only protocols must be amended either using a *Declaration of Emergent Phenotype and Request for a Procedural Change to a Murine Protocol* if originally a paper application, or by direct modification of an approved e-IACUC protocol, whenever aspects of an emergent phenotype, recurring clinical condition(s), or unanticipated outcome are recognized that were not described in the original application (refer to IACUC Principles and Procedures XII.13). Research uses of locally produced unique lines of mice must be described in either an *Application for the Use of Animals in Research* or a separate e-IACUC research protocol, and the produced mice transferred to the approved research protocols using a *Request to Reassign Research Animals* form. Such protocols describing the research uses of locally produced mice must also be similarly amended or electronically modified, as appropriate, whenever aspects of the emergent phenotype, recurring clinical condition(s), or an unanticipated outcome are recognized.

III.8. The e-IACUC draft application must be submitted for veterinary pre-review using the ARC system by the veterinary pre-review submission deadline viewable at http://www3.research.usf.edu/cm/deadlines-schedule.asp. University veterinarians are required to provide applicants with comments and suggestions that may help the IACUC better understand the proposed research involving animals by making direct entries onto the draft e-IACUC application within 7 days of its submission for veterinarian pre-review. Applicants may choose to revise their IACUC application by incorporating changes suggested by veterinarians. Making any addendum, change, or elaboration suggested by the veterinarians does not in any way guarantee or constitute an approval by the IACUC, but should help the IACUC understand better the research plan involving animals.

III.9. Applicants must submit their application in its final form to Research Integrity & Compliance using the ARC system prior to the IACUC submission deadline in order to make that month's IACUC meeting agenda. Submission deadlines to the IACUC are established by the Division of Research Integrity & Compliance, are viewable at http://www3.research.usf.edu/cm/deadlines-schedule.asp , and are dates by which the application must be received in final form. Applications not submitted within sixty (60) days of receipt of the veterinary pre-review are not eligible for inclusion in an IACUC agenda, and to become eligible must be resubmitted for a second veterinary pre-review prior to submitting to Research Integrity and Compliance for inclusion on an agenda. *A Conflict of Interest (COI) Disclosure Form* must also be submitted using the ARC system for each new IACUC application. Applications will not receive full IACUC approval until the *Conflict of Interest (COI) Disclosure* Form has been submitted and a management plan is in place (as appropriate) for each new application and renewal. Information regarding COI and the COI disclosure form are at http://www3.research.usf.edu/cm/subpages/conflict-of-interest.asp.

III.10. The narrative of the PHS funding agency grant, with all approved revisions, that will support the proposed animal use must accompany the protocol application to the IACUC (other than a murine colony only application), as an *electronic-version* uploaded to the e-IACUC application. **The PHS funding agency grant narrative should include the face page**

and research plan of the grant application, which describe the specific aims of the research and the methods involving vertebrate animals, and species of animals that will be used. Protocol applications with other proposed sources of funding (e.g., non-PHS, departmental or corporate) need not be accompanied by a grant proposal, but Comparative Medicine will not fill requests for animals or service under a specific IACUC-approved protocol until an association between fiscal support, an IACUC protocol, and an account or purchase order number is assured in writing. The primary reviewing member of the IACUC must compare the e-IACUC application and the PHS funding agency grant proposal and ensure that they are comparable in scope, (i.e., the protocol represents an aspect of the grant proposal or matches it entirely). Approval of an IACUC protocol application, other than a murine colony only application, with proposed support from a PHS funding agency, is contingent on comparable protocol and proposal titles and narratives.

III.11. The IACUC meeting schedule is viewable at http://www3.research.usf.edu/cm/deadlines-schedule.asp. When additional IACUC meetings are required, reasonable notice is given to the IACUC and public by posting a notice to http://www3.research.usf.edu/cm/deadlines-schedule.asp, at least 72 hours in advance of the special meeting. IACUC members are required to attend each monthly meeting and must notify the IACUC administrative staff of planned absences prior to protocol review assignments.

III.12. New e-IACUC applications are reviewed by the full IACUC membership. Each e-IACUC application is designated a protocol type by the applicant PI as either "research or teaching", "wildlife", "murine colony only", "antiserum production only", or "tissue use only". Protocol types "research or teaching" and "wildlife" are added as an agenda item to the next regular IACUC meeting. Protocol types "murine colony only", "antiserum production only", and "tissue use only" are subject to a designated member review process.

III.13. Protocol types "murine colony only", "antiserum production only", and "tissue use only" are subject to a designated member review process, as follows. Within 3 days of receipt of the new application, any member of the full IACUC can ask for clarifications or revisions of the e-IACUC application involving "murine colony only", "antiserum production only", or "tissue use only", or can call for a review of the application at the next regular IACUC meeting. If full committee review is called, the clarified or revised e-IACUC application is reviewed by the full IACUC. If there are no clarifications, revisions or full IACUC review requests within three days of receipt of the application, the application is reviewed by a designated IACUC member. The designated member reviewer assumes the responsibility for the full committee in granting unanimous approval, requiring modification, or returning the protocol for full review. At any time during this process, any IACUC member may request to view/review the modified application, or may request full committee review of the application. When considered complete and appropriate, the designated member reviewer approves the application, and this approval is communicated to the full IACUC membership at its next regular monthly meeting. Any e-IACUC application that is added as an agenda item to the next regular IACUC meeting, or not approved by the designated member reviewer, is then reviewed by the full IACUC at the next regular monthly meeting. Written IACUC approval is required prior to implementing any animal use.

III.14. Protocol types "research or teaching" and "wildlife" are added as an agenda item to the next regular IACUC meeting. Each new research, teaching, wildlife or other protocol type added as an agenda item to the IACUC meeting is presented by a primary reviewing IACUC member using entries made directly on the e-IACUC application. After verbally presenting their findings regarding the application to a quorum of the IACUC members, the primary reviewing IACUC member proposes a motion to either approve the application, require modifications to secure approval of the application, or disapprove the application. IACUC members listed as participating personnel on the e-IACUC application must leave the room during the presentation and discussion of the protocol and are not permitted to participate or vote. After discussion and a motion is made, the motion is seconded, and the full IACUC committee votes. If the approved motion is "Requires Modification to Secure Approval," it is understood that all members of the IACUC, in attendance or not, forgo a full review of the to-be-revised application in favor of the designated primary reviewing IACUC member's review. Once members have chosen this designated-member review, then the primary reviewer assumes the responsibility for the full committee in granting unanimous approval, requiring modification, or returning the protocol for full review. At any time during this process, any member may request to view/review the modified application, or may request full committee review of the application.

III.15. The applicant PI can annually renew an IACUC-approved protocol for up to a total of two additional one-year renewals, if re-approved by the IACUC. Any changes to the IACUC-approved protocol must be within the scope of the original hypothesis and proposed work, and if originally proposed as a paper application must be described on either a *Request to Amend an Animal Use Protocol* or a *Request for a Procedural Change to an Animal Use Protocol*. If the research was originally proposed as an e-IACUC application, any change must be described by direct modification of the protocol using the ARC system.

III.16. *Amendments* to existing protocols (i.e., a change in the certified research personnel other than PI, title, funding source other than federal sources, addition of another strain of the same species if justified in writing, use of an additional laboratory or relocation of an existing laboratory for an approved activity outside of animal facilities, or the use of

conventional therapeutics, drugs, analgesics, or anesthetics) are reviewed by the IACUC within 7 days of receipt, and approval communicated to the full IACUC membership at its next regular monthly meeting. If an amendment is proposed to a paper IACUC protocol, *A Request to Amend an Animal Use Protocol* form must be submitted in both an electronic and written form which includes the PI's signature. Amendments proposed to an e-IACUC protocol are submitted by direct modification of the approved protocol. Requests to add new research personnel are reviewed first by Research Integrity & Compliance to validate that all documents required of IACUC certification have been completed and submitted via the ARC system. This initial review includes obtaining written confirmation from USF Medical Health Administration that additional health services have been completed, whenever required. Subsequent to this confirmation, the request is reviewed by a designated IACUC member who ensures that all documents required of IACUC certification are complete, that the researcher's profile identifies a new species of interest, and that a current *Health and Risk Assessment for Employee Safety in the Care and Use of Animals* form has been signed by the requesting PI. Requests to change and/or add federal funding agency sources of support (e.g., PHS, NSF and DOD) to an existing protocol other than a "murine colony only" protocol must be accompanied by the grant proposal, with all approved revisions, and submitted as *Procedural Changes* and are subject to full IACUC review.

III.17. *Procedural Changes*, such as requests for additional animals to be made available to the protocol, a change in Principal Investigator familiar with the approved scope of work, or change/addition of a federal agency funding sources for an existing IACUC-approved protocol (other than a "murine colony only" protocol) may be proposed to the IACUC. Such proposals, must be justified in writing, within the scope of the original research hypothesis, and involve the original species. If a change is proposed to a paper IACUC protocol, *A Request for a Procedural Change to an Animal Use Protocol* form must be submitted in both an electronic and written form which includes the PI's signature. The IACUC administrative staff must forward the electronic form of the *Procedural Change* to the full IACUC membership for their consideration. Changes proposed to an e-IACUC protocol are submitted by direct modification of the approved protocol with a justification provided for each change proposed. Procedural changes are subject to full IACUC review.

III.18. *Procedural Changes* are reviewed by the full IACUC membership. Within 3 days of receipt, any member of the full IACUC can ask for clarifications or revisions of the *Procedural Change*, or can call for a review of the *Procedural Change* at the next regular IACUC meeting. If there are no clarifications/revisions or full IACUC review requests within three days of receipt of the revised *Procedural Change*, it is reviewed by a designated IACUC member. If clarifications/revisions are requested, the PI will be given the opportunity to respond and incorporate these changes into the approved protocol. When changes are incorporated, the revised Procedural Change is reviewed by a designated IACUC member. When considered complete and appropriate, the designated member reviewer approves the change, and this approval is communicated to the full IACUC membership at its next regular monthly meeting. Any *Procedural Change* that is added as an agenda item to the next regular IACUC meeting, or not approved by the designated member reviewer, is then reviewed by the full IACUC at the next regular monthly meeting. Written IACUC approval is required prior to implementing any changes.

III.19. *Amendments* and *Procedural Changes* to existing protocols may be communicated to the funding agency at the discretion of the PI. Changes, such as a change in the scope of the original hypothesis, or a change from the original specific aims of the research, or a change in procedures which exceeds the limits set by the IACUC, or a change from the originally proposed species to research, teaching, or testing protocols using animals cannot be amended to an existing IACUC-approved protocol, but must be described on a new e-IACUC application. The PI must obtain prior approval from the funding agency for changes in scope, aims, objectives, or purpose of the research involving animals. These changes in research involving animals and verification of IACUC approval of the newly established protocol must be reported to the funding agency. After three years all continuing studies must be completely re-described in a new application to the IACUC.

III.20. IACUC applications describing mentorship of precollege students are reviewed by the IACUC if the PI attaches all applicable school, county, and/or state forms, including parental permission, the precollege student is certified for animal use within the PI's laboratory, and the PI assures that they or their designated staff who are named on the application will continually directly supervise the precollege student while an animal care staff member is available in the facility during the conduct of the proposed research involving animals.

III.21. IACUC applications describing teaching or training laboratories must assure the IACUC in writing, under whichever Section of the Application is deemed appropriate, that they or their designated IACUC-certified staff who are named on the application will continually directly supervise the students attending the laboratory, and that prior to the lab commencing, will discuss with the students the potential risks and hazards associated with their involvement in the laboratory involving animals, and will document this discussion by having all attending students complete a *Student Safety in Teaching Laboratories Involving Animals* form which the PI will sign and provide to Comparative Medicine before the lab commences.

III.22. All live vertebrate animals used in research, teaching, or testing at the University, or at University-affiliated hospitals or institutes, or by University faculty at other sites other than studies of wild animals in natural settings, must be procured by the Division of Comparative Medicine, and housed and cared for within one of the AAALAC-accredited animal facilities

managed by Comparative Medicine, unless declared in writing in response to item 6.1.2 of the e-IACUC application and approved by the IACUC. Procurement of cryopreserved mouse embryos, oocytes, or spermatozoa from Mutant Mouse Regional Resource Centers or other extramural sources can be arranged by the PI, which informs Comparative Medicine of the request and arrival of these biologics. When described in an IACUC-approved protocol, these can be used or implanted in surrogates housed in quarantine, which are subject to health evaluations prior to reassignment or relocation.

III.23. The University IACUC does not regulate activities involving animals conducted wholly by non-University personnel, off campus, and not supported by University funds. However, in order to document that appropriate practices of acquisition, use, and disposition of vertebrate animal tissues are followed, and with appropriate consideration of occupational health and safety, and public relations issues associated with their use, an e-IACUC "tissue use only" application must be submitted whenever vertebrate animal tissues are requested from another institution, or IACUC-approved protocol, or other source. These requests for the use of animal tissues are reviewed by the IACUC using the designated member review process.

III.24. Use of tissues from animals euthanatized for other purposes or derived from other sources (i.e., slaughter-house, or biological supply houses) reduces the overall number of animals used in research and teaching, and is encouraged by the IACUC. When working under an IACUC-approved "tissue use only" protocol , animals may not be euthanatized for the sole purpose of deriving the tissues, and no protocol-specific ante mortem manipulations may be part of the protocol. Tissues may only be derived as a by-product from animals euthanatized under other IACUC-approved protocols, or from other sources (e.g., slaughter-house tissues).

III.25. Inter-institutional collaborations involving animal use have the potential to create ambiguities about the responsibility for animal care and use, and IACUC oversight. If activities involving animals are to be conducted at another institution involving University personnel, or supported by University funds, there should be a formal written understanding (e.g., a contract, memorandum of understanding, or agreement) which specifies responsibility of offsite animal care and use, animal ownership, and IACUC review and oversight. When applicable, this written understanding should indicate that the collaborating institution has an assurance on file with the PHS, that its program for animal care and use is accredited by AAALAC International, that their IACUC has approved the proposed animal use, and that the USF IACUC will be informed of any issues, concerns or verified noncompliance related to the activity conducted at the collaborating institution. In order to ensure clear definition and understanding of the planned collaboration, whenever the collaborating institution has agreed to perform a significant portion of the animal use aspects of a research grant or contract awarded to the University, the University's IACUC should be provided with written evidence that the collaborating institution's IACUC has approved the activity. These documents must be received from the collaborating institution prior to initiating any work. In addition, the IACUC must be informed of any issues raised by the collaborating institution's IACUC during their inspection of the activity, program, or facility while hosting the research activity. Although the IACUC does not review the animal use aspects of custom commercial polyvalent or monoclonal antibodies by commercial vendors, whenever PHS funds are used to arrange for such, the vendor must have an assurance on file with OLAW and should provide a copy of such to the PI, and if an international vendor, is subject to USDA importation regulations. The IACUC recognizes that wild vertebrate animals owned, housed and cared for by institutions (e.g., zoological parks, aquariums) neighboring the university and its affiliates may, on occasion, benefit from the emergency animal medical diagnostic or therapeutic capabilities of the animal program and its facilities. To ensure that all such emergency animal medical inter-institutional collaborative efforts are appropriately conducted, a formal written understanding (e.g., a contract, memorandum of understanding, or agreement) should be established in advance by the owner of the hosting facility (e.g., CAMLS) with the neighboring institution (e.g., zoological park or aquarium), which specifies responsibilities of animal care and ownership, mechanisms of IACUC reporting, and the source(s) of equipment, supplies, and professional technical support of diagnostic or therapeutic procedures.

III.26. Animals not described in an IACUC-approved protocol are not permitted within University animal facilities, or University research, teaching, or diagnostic laboratories.

III.27. The title and narrative (i.e., specific aims, research plan and methods, and species of animals to be used) of an IACUC application (other than a murine colony only application)must match the title and narrative of the PHS funding agency grant or contract that supports the proposed research activity involving animals. If an activity involving animals that is described in an IACUC-approved protocol is supported by multiple grants with different titles (e.g., one research grant, one postdoctoral fellowship, and departmental funds), or a grant is awarded later during the 3-year approval period of the IACUC protocol that supports the approved activity involving animals (i.e., grant not funded during year one of the protocol, but awarded during year two), the PI must inform the IACUC by modifying the e-IACUC protocol. A single IACUC protocol (other than a murine colony only application) cannot be used to represent the animal use activities of multiple PHS agency research grants or contracts each with budgets to purchase, maintain, and use animals. A single research grant or contract may be represented by more than one IACUC-approved protocol.

III.28. If test substance(s) that are potentially hazardous are to be administered to animals, prior authorization of use of the test substance(s) by the appropriate Safety Committee is required before approval by the IACUC. Approval of the IACUC application involving hazardous materials is contingent on a pre-performance meeting involving the PI and relevant staff that represents the applicant's laboratory, the Division of Comparative Medicine, the IACUC, and the appropriate Safety Committee(s). This pre-performance meeting is required in order to ensure that all involved personnel are aware of the precautions, containment practices, facilities, protective devices, disposal and decontamination procedures, and other necessary safety procedures that must be followed to protect personnel, and prevent accidental animal exposure to the hazardous material. The IACUC may also require a pre-performance meeting whenever an applicant PI proposes infrequently used species or techniques, or proposes surgical or teaching procedures involving anesthetized non-rodent mammals. This pre-performance meeting may take place before or after IACUC approval of the protocol at the discretion of the IACUC, but must occur before initiation of the IACUC approved activity, and ensures that appropriate personnel, equipment, supplies, recordkeeping, and practices of animal care and use have been identified and will be employed.

III.29. The IACUC, assisted by the staff of the Divisions of Research Integrity & Compliance and Comparative Medicine, conduct periodic audits of active animal use protocols, and inspect laboratories outside of the animal facilities where animals are used. These audits and inspections serve as an additional review of the effectiveness of the animal care and use program, and are initiated during each semi-annual inspection of facilities and program by the IACUC, or whenever necessary. These audits and inspections ensure that sufficient animal care and clinical oversight is provided and recorded, that animal pain, distress, or discomfort are anticipated, avoided, or alleviated, that work areas are uncluttered and adequately decontaminated, that current supplies and procedures are used, that appropriately sanitized/sterilized instruments are used, and that the risks of all hazards are minimized. In determining which protocols to audit and laboratories to inspect, the IACUC is especially interested in ensuring the good practices of protocols involving Pain Category C procedures, survival surgery, the administration or use of hazardous materials, or the use of controlled substances.

III.30. The IACUC reviews the animal care and use program and inspects animal facilities, and laboratories outside of facilities where animals are used at least every 6 months. During these reviews and inspections the Semi-Annual Program and Facility Review Report is completed. Deficiencies identified are classified as either "major" (i.e., those which affect animal welfare), or "minor", and a schedule by which corrections will be accomplished is assigned. Each semi-annual report is reviewed and signed by a majority of IACUC members, includes any dissenting views or opinions, and is submitted to the Institutional Official for his review and signature. Each report includes changes made to the program and an itemization of improvements that need to be made to the facilities. Comparative Medicine provides a draft of responses to deficient items to the IACUC Chairperson who in turn provides the Institutional Official with a detailed written assurance of how all items of noncompliance were resolved in a timely manner, when so accomplished. IACUC members must attend and contribute to inspections and must notify the IACUC administrative staff of planned absences prior to an inspection as assigned by the Chairperson. The Institutional Official can dismiss IACUC members and alternates that do not contribute to inspections.

III.31. Non-members of the IACUC that attend IACUC meetings must sit quietly away from the table, and are not allowed to participate in discussions unless questions are directed to them by the Chairperson. Questions regarding animal care and use can be submitted to USF Health Sciences Office of Communications, Public Affairs Office at telephone 974-3300, or fax 974-5422.

IV. Division of Research Integrity & Compliance and Research Grants Regarding Animal Care and Use

IV.1. The Division of Research Integrity & Compliance provides the IACUC with clerical support services, and assists the IACUC with its functions of oversight and evaluation of the animal care and use program.

IV.2. Research Integrity & Compliance maintains both paper and electronic files of IACUC protocols and modifications, and notifies applicants of IACUC actions. Principal Investigators developing applications for VA funded Merit Review grants, which are reviewed and approved by the VA Central Office, must submit the animal component for their research project to the IACUC using the VA Animal Component entitled "*Animal Component of Research Proposal*" (ACORP) viewable at http://www.research.va.gov/programs/animal_research/. All ACORP applications to the IACUC will be assigned a primary reviewer that is a VA resident scientist or administrator familiar with the ACORP format and a regular member or alternate member of the IACUC.

IV.3. Applicant PIs must respond to all communications from Research Integrity & Compliance regarding IACUC deliberations promptly and no later than the designated time frame in the communication. Failure to respond within the designated time will result in Research Integrity & Compliance informing the IACUC of this non-response and recommending administrative closure of the study or pending application.

IV.4. Research Integrity & Compliance maintains research personnel IACUC-certification files, which contain the curriculum vitae of each principal and secondary investigator, and records documenting the training and experience of all research and animal care staff members to ensure that all involved personnel have adequate knowledge and ability to perform their duties of animal care, use, and treatment.

IV.5. The applicant Principal or Secondary Investigator must have a faculty appointment with the University or an appropriate appointment with the James A. Haley Veteran's Administration Medical Center, H. Lee Moffitt Cancer Center and Research Institute, or New College of Florida.

V. Division of Comparative Medicine

V.1. The Division of Comparative Medicine serves as an advocate for all animals housed at University facilities, and implements and administers the AAALAC-accredited Animal Care and Use Program.

V.2. Comparative Medicine provides veterinary oversight of animal health and well-being, guidance and assistance with veterinary medical and surgical techniques, services of disease surveillance, diagnosis and treatment, animal husbandry and nutrition, zoonosis control, hazard containment, and sanitation.

V.3. Comparative Medicine provides supplies, and minor portable equipment, and coordinates the maintenance, repair, replacement, and certification of minor portable equipment.

V.4. The Director of Comparative Medicine has management and administrative authority regarding all aspects of animal care and use, including the procurement, placement, management, husbandry, monitoring, use, and movement of animals involved in research and teaching, and regarding the use of all rooms, areas, and equipment within animal facilities on the University campuses, and at University-affiliated hospitals or research institutes.

V.5. Although veterinary, animal care, and husbandry staff may make contributions to research protocols involving animals, the PI and associated research staff named on the IACUC-approved protocol serve as the primary attending clinicians of all animals housed on behalf of that protocol. As such, the PI must provide coordinated planning, clear direction, and prepared leadership for all protocols under their direction. The PI must ensure that all practices of animal care and use meet or exceed the requirements of applicable principles and procedures and are in accordance with current established veterinary medical and nursing procedures. The PI and research staff are responsible for providing adequate clinical oversight, and post-operative or post-procedural care of the animals, for anticipating and alleviating animal pain or discomfort whenever possible, for identifying the earliest possible clinical endpoint that contributes to the specific aims of the research, and for maintaining complete animal medical records, with entries made in sufficient detail and at intervals specified by these IACUC Principles and Procedures.

V.6. Current USP grade pharmaceutical and biological products must be used in animal therapeutic applications, and in all aspects of animal research whenever available. Use of non-pharmaceutical-grade substances in animals requires written scientific justification to the IACUC, and a written assurance that non-pharmaceutical-grade substance preparation, reconstitution, and/or compounding will result in substances that are sterile, safe, effective, and of an appropriate composition (e.g., purity, concentration, pH, osmolality) and shelf-life, that such preparations will be composed by appropriately trained and experienced personnel, and that adverse animal welfare outcomes related to non-pharmaceutical grade substance use will result in its cessation of use, and a written notification by the PI to the IACUC describing the circumstances of the adverse event.

V.7. Comparative Medicine has authority to establish fee rates for services rendered, to invoice for, and to collect compensation from grant, contract, departmental, or other accounts for services rendered to partially meet the costs of administering the Animal Care and Use Program, and are listed at *Services, Per Diems, Equipment, and Fees*. Comparative Medicine will not fill requests for animals or service under a specific IACUC-approved protocol until an association between fiscal support, an IACUC protocol, and an account is assured in writing. A *Research Services Agreement* and/or a letter on company letterhead indicating a commitment to fund a proposal may be used to document corporate support. A memorandum from the departmental Chairperson or other signature authority on the account may be used to document departmental support of a proposal. The PI must ensure that all costs invoiced to a grant account are allocable to the purpose of the grant. Cost allocable to one protocol may not be shifted to another in order to meet deficiencies caused by overruns, or for other reasons of convenience. Rotation of charges among protocols by month without establishing that the rotation schedule credibly reflects the relative benefit to each protocol is unacceptable. Comparative Medicine will also not honor requests for animals or services under an IACUC-approved protocol until all pre-performance safety and logistical meetings have occurred and been documented in writing by Comparative Medicine.

V.8. Population density and housing space can affect animal reproduction, metabolism, immune responses, and behavior. Comparative Medicine ensures that all standard operating procedures are in accordance with the animal housing space recommendations given in the *Guide*, viewable at http://www.nap.edu/catalog.php?record_id=12910 with the following variances. Static microisolator caging for mice provides 63 in^2 of floor space, sufficient for four adult mice, or one female with nursing litter and her monogamous male mate, or five mice that are each <25 g body weight. Individually ventilated caging (IVC) microisolator caging for mice provides 79 in^2 of floor space, sufficient for five adult mice each >25g body weight. Males of aggressive strains (e.g., BALB/c, SJL, and FVB) may need to be housed at even lower densities. Mice may be bred in a male to female ratio of 1:1, 1:2, or 1:3. If mice are bred in a 1:1 monogamous ratio, the male and female breeders and their litter of pups may be kept together continuously until the pups are weaned. Given the strong postpartum estrous, and relatively short 21 day gestation and 21 day lactation of mice, a female that is continuously housed with a male may be pregnant with her next litter while nursing a current litter. Consequently, pups of continuously housed monogamous breeders must be weaned promptly at 21 days of age, or whenever the subsequent litter is born, whichever is earliest. If mice are bred in a 1:2 or 1:3 harems, each female must be removed to a separate cage when observed to be pregnant, except when nursling mice are euthanatized promptly by postnatal day 7 (e.g., as a source of cells or tissues), in which case the harem may be housed continuously together. Weanlings are counted as adults at 28 days of age or at weaning, whichever is earliest. Whenever Comparative Medicine staff find overcrowded mouse microisolators with occupants that exceed these limits, the cages are flagged by a notice on the cage and an email is sent to the principal investigator and/or research staff immediately and, if no response, 24 hours later. The research laboratory must correct housing densities of mice that exceed the limits set herein within 48 hours, or Comparative Medicine will separate the animals as deemed appropriate, and a charge will be made to the principal investigator's research account.

V.9. Typical HVAC system performance in Florida, when providing the Guide’s recommended fresh-air changes per hour, may result in an occasional relative humidity reading that exceeds the Guide’s recommended humidity range for brief periods in an individual housing room (i.e., less than 10 consecutive days). Facility managers should notify the Assistant Director whenever relative humidity readings are less than 20% humidity or greater than 80% humidity for a period of 10 days or more. Facility Managers should also notify the Assistant Director whenever relative humidity readings are less than 15% humidity or greater than 85% humidity for more than 2 days. The Assistant Director will notify physical plant staff and ask that they evaluate HVAC system performance. If relative humidity readings remain out of range one week after notifying physical plant, the Assistant Director will ask the PI to consider whether these conditions, or the use of portable humidifiers/dehumidifiers or air conditioners, introduce variables that may affect the integrity of their research.

V.10. Prior to departure of employment, faculty must complete an out-processing checklist with the Assistant Director, which delineates the affected IACUC protocols, describes the final disposition of animals, the disposition of any PI-owned equipment and supplies, ensures that all costs will be covered, and ensures that access to animal facilities has been withdrawn.

VI. Preclinical GLP Studies

VI.1. Preclinical studies evaluating medical devices, investigational human or animal drugs, biological or electronic products, or food additives that support or are intended to support applications for research or marketing permits for products regulated by the Food and Drug Administration (FDA) should be conducted in accordance with *Title 21 Code of Federal Regulations Part 58 Good Laboratory Practice for Nonclinical Laboratory Studies* (GLP Study) viewable at http://www.access.gpo.gov/nara/cfr/waisidx_99/21cfr58_99.html .

VI.2. Applicants must declare in the e-IACUC application whether a proposed study will be conducted in accordance with *21 CFR 58* regulations, and attach a copy of the GLP Study protocol generated jointly by the GLP Testing Facility and study sponsor.

VI.3. The PI and GLP Testing Facility management must contact Comparative Medicine and declare in advance their intention to perform a GLP Study, and receive written approval of the GLP Study protocol from the Director of Comparative Medicine, as well as approval of the matching IACUC protocol from the IACUC before initiating any work.

VI.4. The GLP Study Director and QAU must be identified by the GLP testing facility management, and all provisions regarding record-keeping, auditing, data archiving and retrieval, test instrument calibration, and test article identification, handling, and storage accomplished or described in the GLP Study protocol in accordance with *21 CFR 58*.

VI.5. The final report and all data resulting from the GLP Study must be filed with the GLP Testing Facility.

VII. Occupational Health and Safety

VII.1. Health Administration and an occupational health physician offer to all personnel who will be working with animals, information regarding health monitoring, potential zoonoses, and health assessments and immunizations relating to their animal contact and/or exposures.

VII.2. New research and animal care personnel must submit a completed *Health and Risk Assessment for Employee Safety in the Care and Use of Animals* form to the ARC system. Every three years, all IACUC-certified personnel must provide a revised *Health and Risk Assessment for Employee Safety in the Care and Use of Animals* form. Personnel whose duties require access to an animal facility but whose duties do not include working with animals must submit a completed *Personnel Entering Animal Facilities Health and Risk Assessment* to Comparative Medicine at least triennially.

VII.3. Individuals determined to be at risk as determined by completion of the *Health and Risk Assessment for Employee Safety in the Care and Use of Animals* form are offered the opportunity to complete a *Health History Assessment* form and consult the occupational health physician. When special considerations (e.g., use of nonhuman primates) or clinical conditions require additional health services, Research Integrity & Compliance ensures that at-risk personnel make arrangements for services at USF Medical Health Administration and/or the occupational health physician, as required. When additional health services are required, USF Medical Health Administration provides Research Integrity & Compliance with written confirmation that health services have been completed, documentation of which is uploaded to the researcher's profile in the ARC system.

VII.4. Information regarding potential zoonosis and practices of personnel hygiene which limit exposure and risk of contracting zoonosis are provided. The nature of noxious, toxic, hazardous, infectious or carcinogenic agents or compounds when used are posted on the door of the room containing the animal collection exposed to such agents.

VII.5. Individuals are expected to notify their supervisor of suspected health hazards. Whenever work related accidents, injuries, or illnesses occur, individuals are required to report these to their supervisor.

VII.6. Vigilance for zoonoses is an important aspect of any protocol involving human and other xenograft transplantation. The PI must consider the potential for exchange of infectious agents between natural and foreign hosts, including the potential for inapparent infection, when planning or conducting research-involving xenotransplantation. The PI must propose in writing to the IACUC the appropriate precautions that will be instituted regarding health characterizations of the donor, monitoring of the recipient animal, and any handling, management, and containment practices of the biologics, graft, or recipient animal that will be used for a sufficiently long period of time to minimize the risk that pathogens may arise and pose a hazard to personnel or other animals.

VII.7. Animals administered uncharacterized primary tumor resections, tissue explants, blood, or other patient-derived xenografts are housed in ABSL-2 containment in accordance with SOP 408.

VII.8. Research and animal care personnel must consult the occupational health physician whenever research is proposed that involves unvaccinated or uncharacterized carnivores, pregnant sheep, goats, or nonhuman primates.

VIII. Certifying Adequacy of Personnel Training and Experience

VIII.1. All personnel must have adequate knowledge and experience to perform their duties of animal care, use, and treatment.

VIII.2. All personnel must be sufficiently familiar with the AWA, PHS Policy, Guide, and these IACUC Principles and Procedures, so that their care, treatment and use of animals will be in accordance with these principles.

VIII.3. All personnel involved in live vertebrate animal use, treatment, or care must be certified by the IACUC as qualified to perform their duties, and named on the IACUC protocol application(s) to which they will contribute. The IACUC oversees and evaluates the effectiveness of the training program at least semi-annually. All program personnel training must be documented.

VIII.4. Every three years, all research personnel must complete the AALAS Learning Library course entitled "*Laws, Regulations, Policies, and the Guide – USF Orientation Lessons*" and provide the certificate of completion, which documents that they have received training in animal care and use legislation, IACUC function, ethics of animal use and the concepts of the Three Rs, methods for reporting concerns about animal use, occupational health and safety issues pertaining to animal use, animal handling, aseptic surgical technique, anesthesia and analgesia, and euthanasia.

VIII.5. New Principal and Secondary Investigators, and other research personnel with a significant history of work with the species requested, which has been documented by the publication of multiple, peer-reviewed reports of research involving the requested species, are required to provide a copy of their curriculum vitae to document this prior experience.

VIII.6. All new personnel using live vertebrate animals must complete a *Health and Risk Assessment for Employee Safety in the Care and Use of Animals* form. Every three years all research personnel must complete a new *Health and Risk Assessment for Employee Safety in the Care and Use of Animals* form.

VIII.7. All new personnel using live vertebrate animals must complete the appropriate orientation form. Personnel requiring access to an animal facility must complete the *Facility Orientation of Research Staff* form and a *Request for Facility Orientation and Certification of New Research Personnel Using Animals* form by contacting the local facility manager so that a general orientation and protocol-specific, and/or species-specific training can be ensured or arranged. The *Request for Facility Orientation and Certification of New Research Personnel Using Animals* form is left with the facility manager. Personnel planning studies of wildlife in natural setting must complete *an Orientation of Field & Wildlife Research Staff* form. Personnel planning studies that only use animal tissues in areas outside of animal facilities must complete an *Orientation of Research Personnel Using Tissues Outside of Animal Facilities* form.

VIII.8. To become eligible for IACUC certification, all new research personnel must provide four completed documents to Research Integrity & Compliance:

a) *Facility Orientation of Research Staff* form, or *Orientation of Field & Wildlife Staff* form, or *Orientation Research Personnel Using Animal Tissue Outside Facilities* form),
b) *Health and Risk Assessment for Employee Safety in the Care and Use of Animals* form,
c) Certificate of completion for the AALAS Learning Library course entitled "*Laws, Regulations, Policies, and the Guide – USF Orientation Lessons*", and
d) Curriculum vitae or resume.

VIII.9. Comparative Medicine provides training to researchers conducting surgical procedures as requested by research faculty or staff, or required by the IACUC, to ensure that good surgical technique is practiced, including, asepsis, gentle tissue handling, minimal dissection of tissue, appropriate use of instruments, effective hemostasis, and correct use of suture materials and patterns. Attendance and curricula are documented.

VIII.10. Comparative Medicine offers formal hands-on wet-laboratory training. Curricula may vary depending on need as requested by research faculty or staff, or as required by the IACUC, and may include basic animal needs, proper animal handling, routes and methods of substance administration, proper pre-procedural and post-procedural care, aseptic surgical technique, methods of anesthesia and analgesia, use of equipment, and methods of euthanasia.
Attendance and curricula are documented.

VIII.11. Pre-performance meetings of protocols conducted in accordance with *IACUC Principle III.28* must ensure and document that:

a) personnel contributing to protocols involving hazardous material have been/are provided safety training, clearly defined procedures, appropriate personal protective equipment, understand the hazards involved, and are proficient in implementing required safeguards,
b) personnel contributing to protocols involving surgical procedures conducted in nonrodent mammals have been/are provided training in good surgical technique, including, asepsis, gentle tissue handling, minimal dissection of tissue, appropriate use of instruments, effective hemostasis, and correct use of suture materials and patterns,
c) personnel contributing to protocols involving infrequently used species or techniques have been/are provided training in appropriate practices of animal care and use, recordkeeping, and use of appropriate supplies and equipment.

VIII.12. Comparative Medicine provides training and/or coordinates and provides technical assistance with species-specific or protocol-specific techniques whenever new or infrequently used species or techniques are introduced or re-introduced to campus, or additional individual instruction is requested by research faculty or staff, and/or required by the IACUC.

VIII.13. Research personnel intending to request, possess, or use any controlled substance in research or teaching involving animals must also first register with Comparative Medicine c/o the Assistant Director by completing a Certification of Research Personnel Using Controlled Substances form in accordance with *IACUC Principles and Procedures* XIV.2.

VIII.14. Research personnel are kept apprised of updates that reflect changes in Standard Operating Procedures, IACUC Principles, processes, technology, legislation, and other relevant areas by transmitting such updates by email to all IACUC

certified faculty and staff, and by posting such updates at http://www.research.usf.edu/cm/current-topics.asp or http://www.research.usf.edu/dric/iacuc/.

VIII.15. All research personnel are responsible for annually reviewing their personnel profile in the ARC system, and declaring any updates or changes, including reviewing and updating as appropriate their *Health and Risk Assessment for Employee Safety in the Care and Use of Animals.*

VIII.16. The IACUC annually re-certifies all research and animal care personnel as to the adequacy of their training and experience using animals. A change in the certified personnel contributing to an IACUC approved animal use must be declared in advance by the PI and approved by the IACUC prior to such personnel making contributions to protocol(s), using processes delineated in IACUC Principle III.16.

VIII.17. Individuals who comply with these *Principles and Procedures* will be granted an ARC registration number by the IACUC c/o Research Integrity & Compliance to be used in applications for use of animals in research. Research staff or other individuals involved in animal care and use activities suspended by the IACUC can have their certifications revoked by the IACUC. Individuals without a current certification number can be denied access to the services and facilities of Comparative Medicine.

VIII.18. Principal Investigators can request that visiting scientists be granted escorted hands-on access to animals and/or animal facilities in order to collaborate, assist, or instruct in the PI's IACUC-approved activities by providing the IACUC chair via email at iacuc@research.usf.edu with (1) a memorandum naming the visiting scientist, naming the institution from which he/she originates, identifying the dates of their planned presence within facilities, and assuring that the PI or their designated IACUC-certified staff will continually escort the visiting scientist during the conduct of activities involving animals, and (2) a letter on letterhead from the visitor's originating institution which documents that the visiting scientist originates from an institution with an OLAW assurance or an AAALAC accreditation if applicable, and that the visiting scientist is certified for animal care and use activities by the originating institution's IACUC. Additional documentation of completion of health care services by the visiting scientist may be requested by the IACUC when considering such requests for access to animals and/or animal facilities. Once the required documentation is reviewed and approved by the IACUC Chair or his designee the visit can proceed.

VIII.19. All permanent animal care program staff are required to be certified by the IACUC, to prepare for and receive certification by the American Association for Laboratory Animal Science (AALAS), and may make direct technical contributions to approved protocols as stipulated in IACUC Principle V.5 when assigned by Comparative Medicine management. To ensure appropriately trained and experienced animal care staff are assigned protocol-specific duties, Comparative Medicine management maintains a table of degrees and certifications earned (CMDC 184) and a log of wet-lab species-specific trainings and documented proficiencies in methods of surgical, anesthetic, and technical procedures (CMDC 183) for each of its staff.

IX. Reporting Deficiencies in Animal Care or Treatment

IX.1. Reporting concerns, deficiencies, or observations made regarding the adequacy or appropriateness of the facilities, program, principles, or procedures contributes to the oversight, development, and improvement of the program for animal care and use, and contributes to the resolution of the concern or deficiency.

IX.2. Deficiencies in animal care, use, recordkeeping, or treatment, and adverse events in animal care or use must be reported, and can be reported to Comparative Medicine veterinarians (745-4361, 974-4935, 974-3836, or administrative staff (974-9842, 947-9876), or to the IACUC c/o Research Integrity & Compliance (974-0954, 974-3234, 974-7106), or directly to the IACUC Chairperson (974-1547), or IACUC Vice Chairperson (974-1549), or to the Institutional Official of the Animal Care and Use Program, (974-5570).

IX.3. This reporting-feedback mechanism of observations made regarding the practices of animal care and use within these laboratories, contributes an important oversight, assists in the continuous development of the animal program, and includes a mechanism for anonymity viewable at https://secure.ethicspoint.com/domain/en/report_custom.asp?clientid=14773 .

IX.4. Such reports, suggestions, complaints, or compliments are made with protection of the reporting individual from any discrimination or reprisal.

IX.5. Reports of concerns regarding animal welfare are immediately forwarded to Comparative Medicine for assurance by the veterinarians that a concern for animal welfare is not ongoing. A veterinarian can interrupt any activity that jeopardizes the welfare of an animal.

IX.6. All reports of alleged animal welfare concern, deficiency, or noncompliance are forwarded to the IACUC c/o Research Integrity & Compliance. The subject Principal Investigator is informed of the allegation in writing by the IACUC Chairperson and may be asked to respond in writing and/or invited to meet with the IACUC to respond to questions regarding the alleged deficiency.

IX.7. The IACUC deliberates regarding the reported alleged deficiency at its next regularly scheduled monthly meeting, or the IACUC Chairperson can call an emergency quorum to discuss the issue in advance of a regular meeting if deemed necessary. The IACUC Chairperson can choose to invite involved personnel to the meeting to whom questions can be directed. The IACUC reports the findings of its deliberations to the PI and the Institutional Official.

IX.8. Any serious or continuing non-compliance with *PHS Policy*, any serious deviation from the provisions of the *Guide*, and any suspension of any activity by the IACUC is communicated by Comparative Medicine via telephone to the Office of Laboratory Animal Welfare (OLAW) in a timely manner. After IACUC review, institutional resolution, and reporting to the Institutional Official, the Institutional Official submits a formal report describing the circumstances and actions taken to OLAW and other relevant agencies.

X. Suspension and Appeal Process of Animal Use Privileges

X.1. The IACUC may suspend an activity that it previously approved if it determines that the activity is not being conducted in accordance with the AWA, the Guide, or these IACUC Principles and Procedures or when the activity does not match the description of the activity originally approved by the IACUC.

X.2. The IACUC may suspend an activity only after review of the matter at a convened meeting of a quorum of the IACUC and with a suspension vote of a majority of the quorum present.

X.3. If the IACUC suspends an activity involving animals, the PI will be informed in writing of the suspension, its conditions, and the expectations of the IACUC which need to be met before additional activities involving animals resume.

X.4. If the IACUC suspends an activity involving animals, the Institutional Official, in consultation with the IACUC, shall review the reasons for the suspension, take appropriate corrective action, and report that action with a full explanation to USDA/APHIS and PHS/OLAW, AAALAC, and the federal and/or major agency funding that activity.

X.5. In the event that an IACUC-approved protocol expires, or is closed by the PI, or for any reason animals which had been maintained under a formerly approved protocol remain within the facilities, but are not described in an IACUC-approved protocol, the IACUC will serve written notification of non-compliance to the PI, that the subject animals are considered in Comparative Medicine “caretaker status” not available for research use until resolution of non-compliance, and request that the PI either submit a new application for animal use, identify the IACUC-approved protocol which now describes the use of these animals, or formally request in writing that the closed protocol be reopened and reviewed. Absence of a written response to this written notification of noncompliance in excess of thirty days can result in the removal, reassignment, or euthanasia of the subject animals. Requests by the PI for additional animals and/or services will not be filled by Comparative Medicine until all animals are described by an IACUC-approved protocol.

X.6. The PI is responsible for ensuring that adequate fiscal support has been retained, and is available for the procurement, care, and use of all animals registered under their IACUC-approved protocol(s) in their name. The PI must ensure that all costs invoiced to a grant account are allocable to the purpose of the grant. Cost allocable to one protocol may not be shifted to another in order to meet deficiencies caused by overruns, or for other reasons of convenience. Rotation of charges among protocols by month without establishing that the rotation schedule credibly reflects the relative benefit to each protocol is unacceptable. In the event that invoices served by Comparative Medicine to the PI remain unpaid for longer than 30 days after the date of the invoice, accounting in Comparative Medicine will consider said invoices as past due. In the absence of payment, all protocols of the involved PI can be interrupted by the IACUC, and animals can be reassigned, removed, or euthanatized. Requests for additional animals and/or services will not be filled by Comparative Medicine until full payment has been received.

X.7. Since the PI has assured the IACUC on their *Request for the Facility Orientation and Certification of New Research Personnel Using Animals* that the certified personnel contributing to their IACUC-approved protocols has been provided adequate training, and has adequate knowledge and experience to perform their duties, the PI is notified whenever the

performance of their staff is not in accordance with these *IACUC Principles and Procedures*. The PI must ensure that any corrective retraining or actions, required of them or their staff by the IACUC, in response to deficiencies in animal care or use, are accomplished.

X.8. Inadequate animal care or use, inadequate clinical animal oversight or treatment, inadequate anticipation or alleviation of animal discomfort, distress, or pain, inadequate animal medical record keeping, or conducting procedures involving animals prior to their approval by the IACUC can result in the suspension of animal use privileges of the supervising PI and involved research or animal care staff.

X.9. The IACUC deliberates regarding reports of alleged deficiencies at its next regularly scheduled monthly meeting, or the IACUC Chairperson can call an emergency quorum to discuss the issue in advance of a regular meeting if deemed necessary. The IACUC Chairperson can choose to invite involved personnel to the meeting to whom questions can be directed. The IACUC reports the findings of its deliberations to the PI and the Institutional Official

X.10. After its review of the reported alleged deficiencies in animal care or use, the IACUC may elect to require additional training of the involved personnel, and/or may elect to demote the certification status of the involved personnel and/or their PI to probationary status, and/or may elect to suspend or revoke the animal use privileges of that individual and/or PI. Personnel whose certification is placed on probationary status may be required to work only in the presence of another certified staff member, may need to complete additional training, may need to assist with an audit of research and animal medical records, and/or may need to undergo an evaluation of research technique. All such conditions of a probationary certification will be specific as to the consequences of further noncompliance, the length of probationary period, and the conditions that must be met in order to return to full certification status. All actions made by the IACUC regarding the certification status of an individual will be reported to them in writing, with a copy to their supervising PI, and the Institutional Official.

X.11. Subsequent to the decision being made by the IACUC, if the investigator has new information which was not part of the original investigation or there is an issue regarding due process, the investigator may appeal the decision of the IACUC as follows:

- Submit in writing to the IACUC office the issue of concern and position with supporting documents within thirty days of receipt of the suspension letter from the IACUC.

- The IACUC office will acknowledge receipt of the letter in writing and forward the letter and supporting documents to the full IACUC committee.

- The IACUC will discuss the investigator's appeal at a convened meeting with a quorum present. The investigator may request an appearance before the IACUC to present arguments for the reversal of a decision. A written record of the discussion and the meeting will be made.

- Following the discussion at the IACUC meeting, the investigator will be informed of the outcome.

- A summary of the IACUC's re-evaluation/appeal decision will be sent to the investigator and to the Institutional Official. The IACUC office will inform any other institutional or regulatory offices or officials, as appropriate.

- A decision by the IACUC may only be overturned by the IACUC; there is no further appeal process

XI. Transportation, Relocation, or Reassignment of Animals

XI.1. All transportation of animals, including intra-institutional transportation, should occur only when essential since any transit time introduces risks of exposure to environmental extremes, crowding, infectious agents, and possible zoonoses, which can affect animal and public welfare, and the consistency of results. (Refer to *SOP #007 Transportation, Relocation, or Reassignment of Animals*).

XI.2. Transporting of animals to research laboratories, procedural, or testing areas outside of an animal facility may jeopardize the integrity of research data, impairs regulatory oversight, and abrogates the implementation of uniform standards of animal care and use within those areas, and requires strong written scientific or logistical justification the e-IACUC application.

XI.3. Movement of animals within animal housing rooms is discouraged. Movement of animals between animal housing rooms of a single facility, or between separate facilities is discouraged, and permitted only when requested in writing using a *Request to Relocate/Reassign Research Animals* form, and approved by the Assistant Director of Comparative Medicine.

XI.4. Transfer of animals from one IACUC-approved research use (excluding murine production as described in an IACUC-approved murine colony only application, and excluding murine imaging referenced in the originating protocol and described in the Small Animal Model and Imaging Core protocol) to another is permitted only when requested in writing using a *Request to Reassign Research Animals* form, the reassignment is justified in writing, and approved by both the originating and reassigned PIs, and the Program Coordinator. An approved reassignment of animals from one IACUC-approved research protocol to a new IACUC protocol becomes effective on the date of the transfer, and all animal per diem, identifying cards, and inventories are changed to reflect this reassignment. Reassignment of naïve mice originating from an IACUC-approved murine colony only protocol to an IACUC-approved research protocol is accomplished by notifying the facility manager, who completes a *Request to Reassign Locally Produced Mice to a Research Protocol* form, who adjusts all animal per diem, identifying cards, and inventories to reflect this reassignment, which becomes effective on the date of the transfer, and who secures prior approval from the Assistant Director of Comparative Medicine if the reassignment necessitates a physical relocation of mice. Requests for reassignment of non-naïve rodents with respect to major medical, surgical, research or teaching procedures from an approved research protocol to a murine colony only protocol requires IACUC review and approval.

XI.5. Shipment of animals to or from other institutions must be requested in writing using a *Request to Ship Mice To Another Institution*, a *Request to Ship Rats To Another Institution*, or a *Request to Receive Animals From Another Institution* form, approved by the Director of Comparative Medicine or designee, and arranged and accomplished by Comparative Medicine, in compliance with AWA, and the *International Air Transport Association Live Animal Regulations* (IATA 2011).

XI.6. Rodents from unapproved sources are quarantined for five weeks, before their health status and eligibility for release from quarantine is determined. (Refer to *SOP #411 Rodent Quarantine*).

XII. Avoidance and Alleviation of Research Animal Pain and Discomfort

XII.1. The IACUC affirms that the study and use of animals contributes to the advancement of knowledge, and that any potential for research animal pain, discomfort, or distress must be minimized.

XII.2. Pain is a perception of suffering or agony that results from mechanical, thermal, or chemical stimuli acting on nociceptors, which in turn generate impulses via neural pathways, mediating spinal reflexes and central sensory processing by the reticular formation, thalamus, and cerebral cortex. Discomfort or distress is considered to refer to those conditions that are not in themselves painful, but which are disagreeable, and which the animal would normally choose to avoid.

XII.3. Although animals that are in pain may not behave like humans, (e.g., pain in animals may be accompanied by immobility and silence, in contrast to the groans and cries of human patients), it is assumed that procedures that cause pain in humans cause pain in animals.

XII.4. The presence of pain in animals can be recognized by alterations in animal behavior (e.g., reduced activity, reduced grooming, hunched-up posture, altered gait, changes in temperament, vocalizations, reduced food and water intake, reduced urinary and fecal output), and in physiological variables, (e.g., reduced depth of respiration, increased heart rate, and reduced hydration status).

XII.5. Animal pain and discomfort can produce a range of undesirable physiological changes, which may radically alter measured responses to experimental stimuli, as well as the rate of recovery from surgical procedures, hence, its avoidance and alleviation are in the best interest of both the animal and researcher.

XII.6. Reducing post-procedural/post-operative pain and discomfort is accomplished by good nursing care, (e.g., keeping the animal warm, clean, dry and well padded), and by the administration of analgesic drugs.

XII.7. Preemptive and/or postoperative/postprocedural analgesia must be administered whenever procedures are identified that are assumed to produce more than momentary or slight pain and discomfort for an appropriate interval, unless the protocol precludes such practice (Research Pain Category C), the investigator has justified such in writing, and the IACUC has approved such practices. The selection of an appropriate analgesic involves consideration of the level of animal pain anticipated or presumed, the species involved, and the experimental protocol. Severe pain, such as may occur during the post-operative period, can be alleviated by the administration of narcotic analgesics, (e.g., buprenorphine, an opioid partial agonist). Non-steroidal anti-inflammatory drugs, with or without the infusion of local anesthetics, can control

mild to moderate pain, in some species, though is contra-indicated in others. Selection of an appropriate route of administration also involves consideration of the recipient species. For example, oral analgesic drug delivery to rodents (e.g., acetaminophen elixir added to the drinking water of rats) may not afford detectable analgesia.

XII.8. In addition to the avoidance and alleviation of pain and discomfort, adequate post-procedural /post-operative animal care also includes efforts to prevent and/or treat post-anesthetic complications, (e.g., aspiration, hypostatic pneumonia, cardiovascular and respiratory depression, dehydration, and infection).

XII.9. Comparative Medicine maintains an inventory of animal use regarding the potential for pain or discomfort. In their application for animal use, PIs designate the described animal use to one of three categories of research. **Research Pain Category A** involves procedures, which produce momentary, slight, or no pain, discomfort or distress (e.g., unrestrained observation, brief restraint for physical examination, phlebotomy, injection of non-noxious material, tagging/punching of the peripheral ear pinna of mice, and euthanasia using species- and age-appropriate methods described in IACUC Principles and Procedures XX followed by tissue derivation). **Research Pain Category B** involves procedures, which produce more than momentary or slight pain, discomfort or distress, which is alleviated by the use of appropriate anesthetics/analgesics (e.g., surgical or invasive procedures conducted while the patient animal is maintained at a surgical plane of general anesthesia). **Research Pain Category C** involves procedures, which produce pain discomfort, or distress, which cannot, or is not alleviated by the administration of appropriate anesthetics/analgesics (e.g., tumor studies, certain behavioral studies, injection of immunogenic emulsions containing complete Freund's adjuvant, monoclonal antibody ascites production, survival analysis).

XII.10. When proposing Research Pain Category C activities involving animals where painful or stressful outcomes are anticipated or possible, the PI must define in writing the clinical criteria which will be used to ensure timely intervention and treatment, or removal of the animals from the study, either in advance of, or immediately after recognition of the discomfort, or the specific clinical end point at which euthanasia of the animals will be accomplished. The earliest possible clinical endpoint that will contribute to the resolution of the hypothesis must be identified and utilized. If avoidance or alleviation of animal pain or discomfort adversely affects the protocol, the PI must provide a detailed justification of why treatments cannot be initiated. When identifying the earliest clinical endpoint in applications to the IACUC, the PI should consider proposing both early notification criteria (e.g., tumor diameter) which when met causes staff to alert the PI to consider whether study objectives have been met, and also later exclusion criteria (e.g., larger tumor diameter, and/or complications referable to the tumor) which when met requires the euthanasia of the animal. When identifying the earliest clinical endpoint, the PI should refer to section C.2.c. of the ARENA/OLAW Guidebook entitled "Humane Endpoints" viewable at http://grants1.nih.gov/grants/olaw/GuideBook.pdf.

XII.11. The PI and associated research staff should maintain written records of activities whenever painful or stressful outcomes are anticipated or possible. Records should be kept within the animal facility on forms provided by Comparative Medicine, with entries that describe when the painful or stressful outcome is first recognized, what treatments are instituted, and when the discomfort is resolved, or when the animal is euthanatized.

XII.12. The written justification for the use of animals involved in Research Pain Category C procedures must identify and utilize the earliest possible clinical end point that will contribute to the resolution of the hypothesis. If death is determined to be the earliest possible endpoint that will contribute to the specific aims of the research, then a written justification of why an earlier clinical endpoint is inadequate for resolving the proposed hypothesis must be included in the IACUC application. When determining the earliest possible clinical endpoint for a proposed research activity, the applicant PI should refer to the guidelines reviewed in "Humane Endpoints for Animals Used in Biomedical Research and Testing", vol. 41(2), 2000, published by the Institute for Laboratory Animal Research, National Research Council and viewable at http://dels-old.nas.edu/ilar_n/ilarjournal/41_2/ .

XII.13. Investigators must consider the needs and well-being of animals involved in protocols that have a potential to cause animal discomfort, pain, or distress which may not be reliably anticipated or controlled. Such protocols include those of new and/or unique phenotypes of transgenic, knock-out, knock-in, or genetically-engineered rodents. Investigators must provide written assurance of adequate clinical oversight, along with a description of intervention criteria to prevent animal discomfort, or a justification of why intervention conflicts with the proposed investigation. When proposing research involving genetically engineered mice, the applicant PI may find helpful the articles "Welfare Issues of Genetically Modified Animals" published by the Institute for Laboratory Animal Research, National Research Council in Vol. 43(2), 2002, and "*Humane Endpoints for Genetically Engineered Animal Models*" ILAR Vol. 41(2), 2000, each viewable at http://dels-old.nas.edu/ilar_n/ilarjournal/41_2/.

XII.14 Applicant PIs assure the IACUC in their murine colony only protocol that progeny mice will be regularly monitored, that morbidity and mortality records will be reviewed to identify unanticipated health problems, and that the IACUC will be informed immediately whenever recurring health problems develop in colony mice.

XII.15. If, in the course of generating and characterizing genetically-engineered mice, the emergent phenotype includes unanticipated, but recurring health problems, then the original IACUC-approved protocol must be amended using a *Declaration of Emergent Phenotype and Request for a Procedural Change to a Murine Protocol* if originally a paper application or by direct modification of an approved e-IACUC protocol. The request to amend the existing protocol must describe the new health condition(s) that have emerged, must describe the treatments that will be used to alleviate any potential discomfort or justify why treatments interfere with the production and characterization of the colony, and must determine the earliest possible clinical endpoint that will result in the exclusion of the affected animal and its euthanasia.

XII.16. When developing a murine colony only application or amending it, the PI must utilize the earliest possible clinical endpoint(s) that will contribute to the phenotypic characterization of the murine line or genotype (e.g., the onset of disease, or the presence of a palpable tumor must be used as the endpoint, not advanced disease progression, or the development of a particular tumor size or volume). Experimental use of animals produced under an IACUC-approved murine colony protocol must first be proposed to, and approved by the IACUC in a research application. For example, characterizing the progression of a strain/line/genotype-specific pathology or condition, or evaluating whether it is amendable to experimental therapeutic regimes, or evaluating its influence on the incidence of mortality must first be proposed to, and approved by the IACUC in a separate research application, distinct from the murine colony only protocol.

XII.17. Investigators must continuously refine methods so as to avoid, alleviate and/or minimize any animal pain or discomfort associated with experimental protocols, and search for alternatives, which reduce animal pain, discomfort and use.

XII.18. Investigators and their staff must conduct clinical and post mortem investigations whenever animals experience morbidity or mortality not anticipated in the protocol. Investigators and their staff must make entries to nonrodent mammalian medical records, which summarize the clinical diagnostic and necropsy findings of an unanticipated animal morbidity or mortality, which occurs unrelated to the protocol, so that research methods can be refined.

XIII. Anesthesia and Analgesia

XIII.1. In designing the experimental protocol, the applicant investigator is obligated to consult a USF veterinarian regarding research animal issues, including anesthesia and analgesia. The applicant investigator is compelled to institute adequate practices of anesthesia and analgesia. Preemptive and/or postoperative/postprocedural analgesia must be administered whenever procedures are identified that are assumed to produce more than momentary or slight pain and discomfort for an appropriate interval, unless the protocol precludes such practice (Research Pain Category C), the investigator has justified such in writing, and the IACUC has approved such practice.

XIII.2. Guidelines, dosages, and comments regarding historically common anesthetics and analgesics for laboratory animal species can be found in *Guidelines: Anesthesia and Analgesia in Laboratory Animals*.

XIII.3. Ether is both flammable and combustible. Ether inhalation is unpleasant and irritating, can cause profuse bronchial and salivary secretions, coughing, and laryngospasm. Ether inhalation is not an acceptable form of anesthesia within these laboratories, and can be substituted by the use of isoflurane (if an inhalant anesthetic is required for anesthesia), or by other agents, methods, or techniques of anesthesia (note: for substitutes to ether euthanasia, see euthanasia Principles and Procedures below).

XIII.4. Neonatal animals have low energy reserves. Periods of hypoxia can deplete limited hepatic glycogen stores and result in hypoglycemia. Neonates have a reduced capacity to detoxify a wide range of drugs. It is preferable to use inhalational anesthetics so that recovery is rapid and normal feeding is resumed as soon as possible. Although the deliberate production of hypothermia has been shown to produce consistent and safe anesthesia for minor surgical procedures with rapid induction and recovery times in neonatal rodents <4 days of age, inhalational anesthetics should be used in rodents >4 days of age, or during major surgical procedures.

XIII.5. Cooling ectothermic species (e.g., frogs) to 4°C will decrease their metabolism and facilitate their handling, but there is no evidence that whole body cooling reduces pain or is clinically efficacious as an anesthetic.

XIV. Controlled Substances

XIV.1. University Principles and Procedures regarding procurement, distribution, use, security, and record keeping of controlled substances regulated by the Drug Enforcement Administration (DEA) are guided by the regulations detailed in *21 CFR 1300-1308* viewable at http://www.deadiversion.usdoj.gov/21cfr/index.html.

XIV.2. Any faculty member requesting, possessing, or using any controlled substance in research or teaching must be registered with the Division of Comparative Medicine c/o the Assistant Director at MDC 20, phone 974-9876, or fax 974-9432 using a *Certification of Research Personnel Using Controlled Substances form* (refer to *SOP #014 Controlled Substances*). Every two years as part of a program-wide biennial inventory of controlled substances, all personnel possessing or using controlled substance in research must re-certify with Comparative Medicine using a *Certification of Research Personnel Using Controlled Substances* form, and if controlled substance use in research does not involve animals an *Application for the Use of Controlled Substances in Research Not Involving Animals* form.

XIV.3. Registrants must be faculty members, and are responsible for all aspects of these Principles and Procedures. Registrants must identify the controlled substance use in an approved IACUC protocol or an *Application for the Use of Controlled Substances in Research Not Involving Animals*, the individual(s) responsible for assisting in their compliance with these Principles and Procedures, the location where the controlled substance will be securely stored, and ensure that complete records will be maintained. Faculty must ensure controlled substances are stored in an area of limited access securely locked in a substantially constructed cabinet. Controlled substances must be secured behind two locks. Laboratory doors can be considered one lock, if doors of unattended labs are kept locked.

XIV.4. Registered faculty must procure all controlled substances from the Division of Comparative Medicine. Controlled substance distributions to faculty using animals are made through the Facility Manager at the location where animals are housed and used; all other distributions are from the College of Medicine Facility, phone 974-9806, or fax 974-7552.

XIV.5. The University holds and recognizes two institutional DEA registrations for basic and preclinical research protocols (i.e. a Schedule I and a Schedule II-V registration), with the Director of Comparative Medicine as the institutional licensee. All other DEA registrations for use of scheduled substances in non-clinical research are in violation of these Principles and Procedures and must be surrendered to the Division of Research Integrity & Compliance, 974-5638.

XIV.6. Each vial of controlled substance procured under the University's institutional DEA licenses is assigned a unique identifying code that corresponds to that substances Schedule, Federal Drug Code number, and a consecutive vial inventory number.

XIV.7. The Division of Comparative Medicine maintains records of all controlled substance distributions to PIs. These records consist of a chronological log of all controlled substance dispersals indexed by substance and PI.

XIV.8. Request for controlled substances must be submitted in writing to the appropriate manager using the *Supplies, Equipment, and Services Order Form* at least 24 hours prior to being dispensed.

XIV.9. Additional requests for a controlled substance can only be filled when the status of the previous dispersal has been verified with the appropriate manager.

XIV.10. PIs are responsible for maintaining accurate records of controlled substances used while in their possession by recording on the *Controlled Substance Record of Use Log* the amount used, and the amount remaining in each vial. Controlled substances used in USDA regulated species will be recorded in animal's medical record.

XIV.11. PIs are responsible for returning the signed *Controlled Substance Record of Use Log* to the Division of Comparative Medicine when their inventory is depleted. Any unused controlled substance, a controlled substance associated with a completed protocol, an out of date controlled substance, or a controlled substance not represented by appropriate documentation must be forfeited and returned to the Division of Comparative Medicine.

XIV.12. Laboratories, storage cabinets, logs of use, and inventory records are subject to unannounced inspections and audits by the DEA, and unannounced biannual audits by representatives from the Division of Research Integrity & Compliance, and/or Division of Comparative Medicine.

XIV.13. Noncompliance can result in the suspension of privileges to use controlled substances and animals, in accordance with IACUC Principles and Procedures X.2-4.

XV. Surgical Techniques and Post-operative Care

XV.1. Survival surgical procedures involving **any vertebrate species** must be conducted using aseptic technique.

XV.2. **Survival surgical procedures involving USDA regulated species** must be conducted within accredited surgical facilities, where separate areas are provided for pre-operative animal preparation, surgeon's pre-operative scrub, the operating room, and for post-operative monitoring and care of the involved animal(s). The surgeon must wear sterile surgical gloves, gown, cap, and mask when conducting major, survival surgical procedures involving USDA regulated species. Accredited surgical facilities are available within the Surgical Core Laboratory of the College of Medicine and the Center for Advanced Medical Learning and Simulation (CAMLS).

XV.3. Sterile instruments must be used for **all survival surgical procedures** involving any species. Heat sterilization of instruments is ideal. Agents such as chlorine dioxide or gluteraldehydes can be used for cold sterilization. Chlorine dioxide is not documented as being toxic to animal tissue, but it will corrode stainless steel instruments. Glutaraldehyde must be thoroughly rinsed off of instruments with sterile saline or sterile water before use. Instruments must be kept on a sterile drape, pan or tray during the procedures. Soaking in alcohol is not an acceptable means of decontaminating surgical instruments.

XV.4. Preparation of the surgical site for **mammalian survival procedures** should include clipping or shaving the surgical site with enough border area to keep hair from contaminating the incision site. Hair removal should be performed in a room remote from the operating room. The surgical site should be scrubbed at least three times with a germicidal scrub, being careful to scrub from the center of the site toward the periphery. The site can then be rinsed with a 70% alcohol and then painted with dilute tamed iodine solution. Note that alcohol will contribute to hypothermia in rodents if used too liberally. Subsequently, the surgical site should be surrounded with sterile drapes. This not only helps prevent stray hair from entering the surgical field, but also provides an area on which to lay sterile instruments during surgery.

XV.5. For **all vertebrate survival surgical procedures**, the surgeon must wear sterile surgical gloves, a clean laboratory coat, and a surgical mask during survival procedures.

XV.6. **Surgical procedures** involving non-USDA species must be conducted in a designated, uncluttered area, on a bench top or hood surface top, which has been cleaned and treated with an appropriate disinfectant (i.e., Clidox®, Clorox®, Sporicidin®, etc) prior to any procedure. Alcohol as a sole disinfectant of the work surface is not appropriate. (Refer to *SOP #412 Rodent Surgery*).

XV.7. For **all species**, in-date and USP grade anesthetics, analgesics, pharmacologics, and supplies must be used. If volatile anesthetics are used, appropriate scavenging must be in use. Preemptive and/or postoperative/postprocedural analgesia must be administered whenever procedures are identified that are assumed to produce more than momentary or slight pain and discomfort for an appropriate interval, unless the protocol precludes such practice (Pain Category C research), the investigator has justified such in writing, and the IACUC has approved such practice.

XV.8. **Mammalian** body cavities are typically closed in at least two layers, with an absorbable inner layer(s), and a nonabsorbable skin layer, or absorbable subcuticular layer. Holding tissue layers must be sutured in an interrupted pattern. Nonabsorbable skin sutures or staples must be removed 10-14 days post-operatively.

XV.9. **Mammalian** post-operative recovery must incorporate the use of an approved ancillary heat source or in an otherwise warm environment, and under direct supervision until the patient animal has fully recovered from anesthesia (i.e., sternal recumbency and intentional movement).

XV.10. Log entries describing surgical events or procedures requiring a surgical plane of anesthesia involving **USDA** regulated **species** must be kept by the PI, in the animal facility, on a *Surgical Record* form, or *Record of General Anesthesia* form, or *Rodent Surgical/Procedural Record* form (provided by Comparative Medicine). Log entries must include, as a minimum, the following, (A) a pre-operative assessment, (B) an anesthetic plan, (C) records of the induction and of the monitoring of general anesthesia, (D) a brief description of the surgical procedures performed, (E) an intraoperative assessment, (F) a record of recovery from anesthesia (or method of euthanasia while the animal is anesthetized), (G) a post-operative assessment, and (H) any complications, treatments, and/or plans, as requested on the appropriate form.

XV.11. Post-operatively/procedurally, following recovery from a surgical plane of anesthesia, nonrodent mammals must be clinically evaluated (e.g., heart or pulse rate, respiratory rate, mucous membrane color or capillary refill time, and body temperature) and observations recorded by the research staff at least once between post-operative/procedural days 1-3. Daily entries of animal health assessment must be made on post-operative days 1, 2, and 3 for all USDA regulated species. This must be done in the medical log, on forms provided by Comparative Medicine. In addition, the dose and route of all post-operative/procedural analgesics, antibiotics, and treatments, and the date of skin suture removal, when applicable, must be noted.

XV.12. Post-operative and post-procedural care for all **mammalian** species during and subsequent to recovery from

general anesthesia includes appropriate analgesia and nursing care, monitoring physiological functions, monitoring behavior, observing for any complications, and appropriate record keeping. To ensure sufficient post-operative/procedural clinical oversight and patient care, mammals recovered from general anesthesia should remain housed within the facility where the surgery or procedure involving general anesthesia was performed for a minimum of 14 days after the date of the procedure. A *Request to Relocate Research Animals* that lists nonrodent mammals, which have undergone general anesthesia within 14 days of the date requested for animal relocation, must be approved by the Director of Comparative Medicine or designee.

XV.13. **Nonmammalian surgical procedures** are conducted in a designated, uncluttered area, which has been cleaned and treated with an appropriate disinfectant (i.e., Clidox®, Clorox®, or Sporicidin®**)** prior to any procedure. Alcohol as a sole disinfectant of the work surface is not appropriate.

XV.14. The skin of **scaled terrestrial vertebrates** can be prepared with dilute Betadine®, Nolvasan®, 10% chlorhexidine, or 70% ethanol. The skin of **amphibian** and **teleostean vertebrates**, which is delicate and easily damaged, contains glands that produce antimicrobial substances. Consequently, risks associated with the use of topical skin disinfectants with these species outweigh their potential benefits, and their use is not recommended.

XV.15. For **scaled aquatic vertebrates**, surgical site disinfection should be done quickly and gently to avoid irritating the skin in a manner that may delay healing. The incision site is dried and disinfected with a suitable compound. Alcohol-based surgical scrubs are not recommended. Povidone iodine-based disinfectants, especially those which do not include acids and detergent compounds (i.e. Ovidine®, Syndel Laboratories International) can be gently applied to the operative area. No scrubbing, per se, is required, as disinfectants will flow into the remaining mucus and cuticle. Simple drying, using sterile gauze or towels, followed by an application of the disinfectant will usually suffice. Suitable drapes are applied so that only the incision site is exposed.

XV.16. Absorbable suture material used in deep tissue closure for **nonmammalian vertebrates** should be those that are absorbed by hydrolysis (e.g., polydioxanone or polyglactin 910), and not those that rely on proteolysis (e.g., chromic catgut) which induce inflammation and have a prolonged presence postoperatively.

XVI. Multiple Major Survival Surgical Procedures

XVI.1. Major surgery penetrates and exposes a body cavity (e.g., laparotomy, thoracotomy, craniotomy), produces impairment of physical or physiologic function (e.g., joint replacement, limb amputation) or involves extensive tissue dissection or transection.

XVI.2. Multiple major survival surgical procedures on a single animal are discouraged but may be permitted if scientifically justified by the applicant and approved by the IACUC.

XVI.3. Multiple major survival surgical procedures may be justified if they are related components of a research project, if they conserve scarce animal resources, or if they are needed for clinical reasons. Cost savings is not an adequate reason for performing multiple major survival surgical procedures.

XVI.4. If multiple major survival surgical procedures are approved by the IACUC, particular attention must be provided by the research staff to animal health and well-being through frequent and continuing evaluations.

XVI.5. Multiple partial ovariectomies involving *Xenopus* frogs is permissible if scientifically justified, perhaps in part due to the need to identify individual frogs that produce oocytes of sufficient quality for the proposed study. As an example, oocytes from different frogs can have quite different efficiencies of DNA transcription or RNA translation, and this quality is rather constant over time. Consequently, it may be desirable to identify those individual frogs that produce acceptable quality oocytes for the planned transcriptional or translational assays, and reharvest oocytes from those frogs. If such an approach is proposed and scientifically justified to, and approved by the IACUC, no more than 4 partial ovariectomies may be performed on an individual frog, and a minimum 3-week inter-operative interval must be allowed. Records must be kept that identify the individual frogs involved, and the dates and number of surgeries performed on each frog.

XVII. Antiserum Production

XVII.1. When designing an antiserum production protocol, the applicant for animal use is directed to *SOP #023 Production of Polyclonal Antibodies in Rabbits* and reviews of adjuvants and procedures of polyclonal antibody production, such as *Antibodies: A Laboratory Manual*, E. Harlow, & D. Lane, Cold Spring Harbor Laboratories, 1988, and those published by

the National Research Council, National Academy of Science in the Institute of Laboratory Animal Research (ILAR) Journal, volume 37, number 3, pages 93-124, 1995; and volume 46, number 3, pages 241-257 and pages 269-279, 2005.

XVII.2. The following is provided only as a guide in designing an immunization and phlebotomy schedule. Alternative approaches to antiserum production which involve other schedules or adjuvants, or which minimize animal use and discomfort are encouraged. Below, the use of complete and incomplete Freund’s adjuvant, and the Ribi®, and TiterMax® adjuvant systems are described.

XVII.3. Investigators who plan to utilize one of the sample protocols for antiserum production described in CM SOP #023 can submit a modification of the core “*antisera production only*” to the IACUC via Comparative Medicine for e-IACUC approval and in doing so will comply with these Principles and Procedures. This application requests that all administrations, observations, phlebotomies, and treatments of rabbits used for antisera production be made and recorded by staff of Comparative Medicine. The PI who is submitting the modification provides the immunizing antigen to the Manager, 974-9806, who in turn coordinates the service.

XVII.4. Purpose & Procedure for which animals will be used: Polyvalent antiserum, are reagents useful in immunoprecipitation, immunoblotting, enzyme-linked immunosorbent assay, and in other *in vitro* experimental procedures. Rabbits will be used to produce antiserum to antigens (typically proteins, native, polymerized, or linked to carriers). Proteins will be administered to rabbits by intradermal, subcutaneous, or intramuscular injection, blood drawn, sera clarified, the presence of antibodies which react specifically with the antigen detected and quantified, and the antiserum collected by exsanguination.

XVII.5. Characteristics of Animals: Production of antiserum is best accomplished in 2-4 kg female NZW rabbits, because they are genetically divergent from the principle sources of most antigens of interest, (i.e., human and mice), and because adequate volumes of antiserum can be readily collected.

XVII.6. Rationale of Number Requested: Antibody specificity of antiserum can vary between individual animals with respect to the dominant antigenic epitopes recognized on a given antigen, hence 2-4 rabbits are immunized with each antigen, and the resultant antiserum screened for the optimal responder.

XVII.7. Pain Category of Research: Pain Category B. This animal use when using adjuvants other than complete or incomplete Freund’s adjuvants may produce minor pain, discomfort, or distress associated with the rare development of sterile granuloma(s) at the immunization site(s), which is alleviated by the use of appropriate veterinary treatment and analgesics.

XVII.8. Experimental Procedures: Standard housing and routine husbandry and handling practices will be followed. Surgery will not be performed. Substances (proteins and adjuvants) will be administered. Specimens (blood) will be collected ante mortem. No other experimental procedures will be performed.

XVII.9. Laboratory Principles and Procedures: All work will be conducted within one of the vivaria in common animal use rooms. Human patient procedural areas, and other areas outside of the vivaria will not be used. Animals will not be locally produced.

XVII.10.Euthanasia: Animals will be euthanatized by administering either 150 mg/kg Pentobarbital or 1 ml/4.5 kg Euthasol IV, or if requested by the PI, will be deeply anesthetized with 40-45 mg/kg Ketamine and 5-8 mg/kg Xylazine IM, exsanguinated, and death assured by the absence of cardiovascular and respiratory movements. Death, other than death from euthanasia, is not an endpoint.

XVII.11.Test Substances: Adjuvants and antigenic substances will be administered. Adjuvants known to produce less intense inflammatory responses (e.g., TiterMax®, Ribi Adjuvant System® (RAS), Montanides®, Syntex Adjuvant System® (SAF), aluminum compounds, and subcutaneously-implanted chambers) should be strongly considered as alternatives to Complete Freund’s Adjuvant (CFA). Use of Freund’s adjuvants requires written scientific justification. CFA can be used only for the primary immunization, while incomplete Freund's adjuvant (IFA) can be used in the subsequent immunizations. CFA and IFA will only be administered by the subcutaneous or intramuscular routes. Intraperitoneal, intravenous, or footpad injections are not acceptable routes of administration. Alternative adjuvant systems should be considered whenever possible. The Ribi® adjuvant system consists of monophosphoryl lipid A, synthetic trehalose dicorynomycolate, and cell wall skeleton, and recommendations of the manufacturer are followed (Ribi Immunochem Research, Inc., 406-363-6214). The TiterMax® adjuvant system consists of a block copolymer CRL-8941, and recommendations of the manufacturer are followed (Hunter’s TiterMax® 800-345-2987).

XVII.12. For all immunizations, protein antigen in saline (avoid Tris-based buffers) is mixed vigorously with an equal volume of adjuvant to generate a thick emulsion that does not disperse on the surface of saline (avoid plastic syringes).

XVII.13. Induction of an adequate immunologic response to an administered antigen, and hence the production of a useful antiserum, is influenced by the route and dose of administration. For each immunization of a protein antigen, between 50-1000ug of antigenic protein is administered. When sufficient antigenic protein is available, >250ug should be administered per immunization. For each injection, 10-100ug of protein is administered per site.

XVII.14. Maximal volume of antigen-adjuvant emulsion per site, maximal number of sites of administration/animal, the gauge of needle to be used for each route are; for subcutaneous, 200 µl/site at ≤12 sites/animal using a 23-25 g. needle; for intradermal, 100 µl/site at <10-12 sites/animal using a 25 g. needle; and for intramuscular, 500 µl/site at ≤3 sites/animal using a 23-25 g. needle.

XVII.15. Schedule of immunizations and phlebotomies are, preimmune bleed followed by primary immunization on day 0, first immune bleed on day 14, second immunization on day 21, second immune bleed on day 35, third immunization on day 42, third immune bleed on day 56, fourth immunization on day 63, fourth immune bleed on day 77, and fifth immunization if needed on day 84, with the fifth immune bleed on day 98.

XVII.16. Animals are monitored daily, and a log of all administrations, phlebotomies, and observations are kept on forms provided by Comparative Medicine in the animal facility.

XVII.17. Specimen Collection, Ante Mortem: Blood (5-10 ml) is drawn prior to immunizations, and 12-14 days following each immunization. Animals are carefully restrained, and in some cases tranquilized with 1-3 mg/kg Acepromazine IM for phlebotomies. Blood is drawn from the marginal ear veins or auricular artery using a 25 g. needle/butterfly. When an adequate titer is detected, approximately 2.5 ml/lb (5.5 ml/kg) body weight of blood is collected every 14 days as needed. Additional immunizations and bleedings at appropriate intervals that reflect this schedule may be performed to ensure that this animal use results in the production of a high titer of useful antibody. Terminal exsanguination of anesthetized animals by cardiac puncture using an 18 g. needle is accomplished when a sufficient volume of high titer antigen-specific antiserum has been collected.

XVII.18. Alternative adjuvants that may be less irritating and inflammatory, and alternative approaches to antiserum production which involve other schedules or adjuvants, or which minimize animal use and discomfort are encouraged, and must be considered. Requests for animal use to produce antiserum other than as described above are encouraged, and made by submitting a completed application for animal use to the IACUC for review.

XVIII. Monoclonal Antibody Ascites Production

XVIII.1. The IACUC acknowledges that the Institute of Laboratory Animal Research (ILAR) and the Office for Laboratory Animal Welfare (OLAW) have identified advantages of monoclonal antibody production in mice against which *in vitro* technologies must compete, that practical *in vitro* methods exist which can replace the ascites method, and that all requests to use the mouse ascites method need to be critically evaluated.

XVIII.2. It is acknowledged that production is rapid in mice, mice can support the growth of cell lines that are often difficult to culture, the antibody in ascites is highly concentrated, and the requirements for materials, equipment, and facilities is far less. It is further acknowledged that the mouse ascites method of monoclonal antibody production can cause mice discomfort. Alternatives to ascites production of monoclonal antibodies are encouraged.

XVIII.3. When critically evaluating a request to use the mouse ascites method, the applicant must demonstrate in writing to the IACUC that the proposed use is scientifically justified, that methods that avoid or minimize discomfort, distress, and pain (including *in vitro* methods) have been considered, and that the latter have been found unsuitable.

XVIII.4. When considering an application for ascites production of monoclonal antibodies the IACUC considers the request in light of the following guidelines. Ascitic fluid volume will not exceed 20% of baseline body weight. Isoflurane is used as an inhalant anesthetic during abdominal paracentesis. Approximately two milliliters of warm saline or lactated ringers solution is administered SQ following paracentesis to compensate for fluid loss. The third paracentesis is performed post mortem, and only three paracentesis will be performed.

XVIII.5. Animals are monitored daily and weighed regularly for the degree of abdominal distension. Regular (2-3 taps) and timely (every 1-3 days) abdominal paracentesis are required to prevent any animal discomfort (ruffled coat, hunched

posture, pallor, inactivity) in advance of complications, which could evolve. Animals in discomfort are euthanatized. A log of all activities and observations is kept.

XIX. Animal Identification and Medical Records

XIX.1. Adequate animal care includes adequate animal medical record keeping. Although veterinary, animal care, and husbandry staff may make contributions to research protocols involving animals, the PI and associated research staff named on an IACUC-approved protocol serve as the primary attending clinicians of all animals housed on behalf of that protocol. As such, research staff are responsible for providing adequate clinical oversight, and post-operative or post-procedural care of the animals, for anticipating and alleviating animal pain or discomfort whenever possible, and for maintaining complete animal medical records, with entries made in sufficient detail and at intervals specified by these IACUC Principles and Procedures. (Refer to *SOP #012 Animal Medical Records*).

XIX.2. Animals are identified on cage cards as to the requesting PI, the IACUC file #, date of arrival, source, and physical findings, including species, strain, sex, weight or age, and should include any identifying features, and/or permanent markings. (Refer to *SOP #015 Animal Identification*).

XIX.3. Viewable at http://www.informatics.jax.org/mgihome/nomen/index.shtml#mnrg the *Nomenclature Rules and Guidelines* for identifying or naming mouse lines or strains of the International Committee on Standardized Genetic Nomenclature for Mice should be followed.

XIX.4. All animals should be acclimated a minimum of seven days prior to use.

XIX.5. Although an animal medical record may be initially established by Comparative Medicine, it is maintained by the PI and associated research staff. Individual animal medical records of all nonrodent mammals must at least include an *Arrival Status* form that describes the condition and characteristics of each animal upon arrival, and *Progress Notes* forms that record items described in *IACUC Principles and Procedures XIX*., each provided by Comparative Medicine.

XIX.6. Log entries that must be made by the PI and research staff on *Progress Notes* forms provided by Comparative Medicine include describing all procedures, substance administrations, tissue collections, observations, treatments, or uses involving USDA regulated species. Procedures or assessments that are approved by the IACUC must be performed and recorded by the PI and research staff at intervals indicated in the approved protocol. Logs maintained for USDA regulated species must be kept by the research staff in the animal facility.

XIX.7. Medical records of all USDA regulated species must also include weekly entries made by the research staff on *Progress Notes* forms, which at least summarize, an impression of overall condition, food and water intake, and voidings, any clinical abnormalities or complications, any treatments administered in response to observed abnormalities, and any experimental procedures.

XIX.8. Medical records of nonrodent mammalian species, with the exception of nonhuman primates, must include a monthly clinical entry re-characterizing the condition of the animal, conducted by the veterinary and/or animal care staff. Nonhuman primate medical record entries will occur in conjunction with tuberculin skin testing and physical examinations. Entries re-characterizing the condition of non-rodent mammals should include the animal's body weight, body temperature, heart or pulse rate, respiratory rate, and mucous membrane color or capillary refill time, and are logged on *Progress Notes* forms.

XIX.9. When clinical abnormalities are recognized in USDA regulated species, the PI and research staff must make entries in the medical record, which at least document, the abnormal physical/physiological parameters observed, a description of specimens taken for diagnosis, the laboratory/diagnostic findings, and treatment(s) initiated.

XIX.10. Log entries describing surgical events or procedures requiring a surgical plane of anesthesia involving **USDA regulated species** must be kept by the PI, in the animal facility, on a *Surgical Record* form, or *Record of General Anesthesia* form*, or Rodent Surgical/Procedural Record* (provided by Comparative Medicine). Log entries must include, as a minimum, the following, (A) a pre-operative/procedural assessment, (B) an anesthetic plan, (C) records of the induction and of the monitoring of general anesthesia, (D) a brief description of the surgical or other procedure(s) performed, (E) an intra-operative/procedural assessment, (F) a record of recovery from anesthesia (or method of euthanasia while the animal is anesthetized), (G) a post-operative/procedural assessment, and (H) any complications, treatments, and/or plans, as requested on the appropriate form.

XIX.11. Post-operatively/procedurally, following recovery from a surgical plane of anesthesia, nonrodent mammals must be clinically evaluated (e.g., heart or pulse rate, respiratory rate, mucous membrane color or capillary refill time, and body temperature) and observations recorded by the research staff at least once between post-operative/procedural days 1-3. Daily entries of animal health assessment must be made on post-operative days 1, 2, and 3 for all USDA regulated species. This must be done in the medical log, on forms provided by Comparative Medicine. In addition, the dose and route of all post-operative/procedural analgesics, antibiotics, and treatments, and the date of skin suture removal, when applicable, must be noted.

XIX.12. The PI and associated research staff should maintain written records of activities whenever painful or stressful outcomes are anticipated or possible in any animal. Records should be kept within the animal facility on forms provided by Comparative Medicine, with entries that describe when the painful or stressful outcome is first recognized, what treatments are instituted, and when the discomfort is resolved, or when the animal is euthanatized.

XIX.13. Unanticipated clinical abnormalities or complications in any animal must be resolved through the cooperative interaction of research, animal care, and veterinary staff.

XIX.14. Research staff must make entries to the medical records of USDA regulated species that summarize the clinical diagnostic and necropsy findings of an unanticipated animal morbidity or mortality that occurs unrelated to the protocol, so that research methods can be refined.

XIX.15 The final disposition of USDA regulated species must be clearly described in the animal's medical record and filed with the facility manager of Comparative Medicine when completed.

XIX.16. Inadequate animal care or inadequate animal medical record keeping can result in the suspension of animal use privileges in accordance with IACUC X.

XX. Animal Euthanasia

XX.1. Euthanasia is the induction of humane death without pain, anxiety, or distress. Acceptable techniques safely result in rapid animal unconsciousness, cardiac and respiratory arrest, and loss of brain activity.

XX.2. Anxiety and distress can be minimized, and safety assured by careful handling, calming, and gentle restraint, and by the appropriate selection of, and training and experience in euthanasia technique suitable to the research protocol and the species used.

XX.3. Acceptable, conditionally acceptable, and unacceptable euthanasia agents, methods and techniques have been described by the *American Veterinary Medical Association Guidelines on Euthanasia*, 2013, viewable at http://www.avma.org/issues/animal_welfare/euthanasia.pdf . These recommendations provide the basis for acceptable euthanasia techniques and are adopted as such. These recommendations and those in the *Guideline of the American Society of Mammalogists for the Use of Wild Mammals in Research*, the *Guidelines for Use of Fishes in Research*, the *Guidelines for Use of Live Amphibians and Reptiles in Field and Laboratory Research*, and the *Guidelines to the Use of Wild Birds in Research* provide the basis for acceptable euthanasia techniques of wild, avian, aquatic, and ectothermic animals, and are adopted as such. Specific Principles and Procedures regarding historically common euthanasia techniques follow.

XX.4. Ether is both flammable and combustible. Ether inhalation is unpleasant and irritating, can cause profuse bronchial and salivary secretions, coughing, and laryngospasm. Ether inhalation is not an acceptable form of euthanasia, and must be substituted by the use of an inhalant anesthetic, or by other agents, methods, or techniques of euthanasia.

XX.5. Decapitation or cervical dislocation of deeply sedated or anesthetized rodents and other small animals is an acceptable method of euthanasia when justified to, and approved by the IACUC prior to their use. A request to perform decapitation or cervical dislocation without sedation or anesthesia requires a strong written scientific justification explaining the experimental design that necessitates this method of euthanasia. Staff who intend to perform decapitation or cervical dislocation without sedation or anesthesia must have demonstrated competence under the observation of the Comparative Medicine Training Coordinator.

XX.6. Chloral hydrate is only acceptable for euthanasia of deeply anesthetized large animals, and only when administered intravenously. Chloral hydrate is not acceptable for dogs, cats, and other small animals.

XX.7. Chloroform is hepatotoxic, cardiotoxic, and nephrotoxic, is an unacceptable method of euthanasia, and should be substituted with other agents, methods or techniques of euthanasia.

XX.8. Carbon dioxide inhalation is an acceptable method of rodent euthanasia, but is not an acceptable technique with rabbits or larger animals. Compressed carbon dioxide in gas cylinders is the only acceptable source of carbon dioxide for euthanasia and generation of CO_2 by any other means such as dry ice or antacids is unacceptable. Sudden exposure to high concentrations of CO_2 may be distressful to some species. Exposure of rodents to gradually increasing concentrations of CO_2 (i.e., displacement rate from 10% to 30% using an appropriate pressure reducing regulator and flow meter) may help avoid or minimize discomfort or distress. Euthanasia by inhalation of any gaseous agent must be followed by the assurance of the cessation of cardiovascular and respiratory movements by observation at room air for at least 10 minutes, or by employing a secondary method of euthanasia such as cervical dislocation, decapitation, or bilateral thoracotomy. Death must be verified prior to carcass disposal.

XX.9. The intravenous injection of a barbituric acid derivative is a preferred method of euthanatizing rabbits, cats, dogs, ruminants, and swine. Intraperitoneal injection of a barbituric acid derivative is a preferred method of euthanizing rodents and other small animals, such as guinea pigs or hamsters. Adequate pentobarbital euthanasia dosage for all species is ≥ 150 mg/kg.

XX.10. Exsanguination is not used as a sole means of euthanasia. Animals may be exsanguinated when surgically anesthetized.

XX.11. Neonates are resistant to hypoxia. Consequently, inhalants should not be used alone as a sole means of euthanizing neonates. Inhalants may be used to induce unconsciousness, followed by another method of euthanasia to ensure death. Euthanasia of feti or prenatal mice should be accomplished immediately after removal from the dam. Incomplete neural development in mouse feti less than 14 days of gestation suggests that pain perception at this age is minimal.

XX.12. Cooling ectothermic species (e.g., frogs) to 4°C will decrease their metabolism and facilitate their handling, but there is no evidence that whole body cooling reduces pain. Hypothermia prior to physical methods of euthanasia, or hypothermia alone are unacceptable methods of euthanasia.

XX.13. Following all methods of euthanasia, animal death is assured by the determination of the cessation of cardiovascular and respiratory movements.

XXI. Animal Acclimation and Conditioning

XXI.1. Investigators are encouraged to use specific pathogen free animals for research and teaching, and recognize that co-morbidities complicate and interfere with the interpretation of research data.

XXI.2. All animals should be acclimated a minimum of seven days prior to use. Animal conditioning should be implemented whenever animals with unspecified clinical history or health status are requested. (Refer to *SOP #005 Animal Quarantine, Stabilization, and Separation*).

XXII. Animal Facilities

XXII.1. All facilities for University of South Florida animal care and use must be listed on the registration #58-R-0015 with the United States Department of Agriculture, Animal and Plant Health Inspection Service (USDA/APHIS).

XXII.2. Each college, affiliated hospital, or research institute which hosts a facility for animal care and use is responsible for all physical structural maintenance and repairs, and all major equipment maintenance, repairs, and replacements within their respective facilities, including, but not limited to exterior roof, walls and doors, utilities, lighting, heating/ventilation/air conditioning, interior wall/floor/ceiling surfaces and doors, cage washers, sterilizers, boilers, steam generators, water conditioners/softeners, animal water treatment and distribution systems, isolation cubicles, and security/environmental monitoring systems, so as to meet the requirements of the *Guide*, AWA, and the standards of accreditation by AAALAC.

XXII.3. In the event that space within their facilities becomes limited, a host college, hospital, or institute which maintains an animal facility may request that the Director of Comparative Medicine show priority to investigations conducted by their faculty or members, or investigations within their discipline, when assigning space for animal housing, care, and use.

XXII.4. All proposals, plans, and construction documents which are developed in order to create new, additional animal facilities, or to renovate existing animal facilities, must be developed in compliance with the recommendations delineated in the Guide 2011, so that the program for animal care and use managed by Comparative Medicine can be readily implemented and administered in compliance with the Guide 2011, AWA, and the standards of accreditation by AAALAC.

XXII.5. As such, all proposals, plans, and construction documents involving the construction or renovation of space for animal care and use must be reviewed and approved by the Director of Comparative Medicine to ensure that newly constructed or renovated facilities can be readily incorporated into the animal care and use program.

XXII.6. All newly constructed or renovated animal facilities must be incorporated into the animal care and use program administered by Comparative Medicine.

XXII.7. All costs of development and construction or renovation of animal facilities must be met by the host college, hospital, or research institution.

XXIII. Studies of Wild Animals In or From Natural Settings

XXIII.1. The IACUC acknowledges that the federal government, including the National Science Foundation (NSF) requires its grantees to comply with the *Guide* and the *PHS Policy*.

XXIII.2. The *Guide* and *PHS Policy* charge the IACUC with oversight of the experimental procedures, and methods of handling, care, and use of free-living wild vertebrate animals. These activities must comply with the *U.S. Government Principles for the Utilization and Care of Vertebrate Animals Used in Testing, Research, and Training* as outlined in Appendix B of the *Guide*.

XXIII.3. As a federal grantee, the University has filed written assurance #A4100-01 to comply with the *Guide* and *PHS Policy*.

XXIII.4. Studies of wild animals in natural settings (referred to herein as “field studies”) contribute to the conservation and well-being of wild animals. Efforts to protect indigenous animal species often are dependent on an ability to learn which species are present, the nature of their habitat, distribution, ecology, anatomy, physiology, and reproduction. The University affirms that the respectful use and treatment of all animals is both an ethical and scientific necessity.

XXIII.5. The IACUC recognizes that free-living wild vertebrate animals comprise a considerable range of diversity represented by over 20,000 species of fishes, over 8,000 species of amphibians and reptiles, and over 9,000 species of birds, with varied and often poorly known behavioral, physiological and ecological characteristics.

XXIII.6. The IACUC recognizes that field studies often involve many species, some of which may be unanticipated or even unknown to science before the onset of the study.

XXIII.7. The IACUC recognizes that it is not always possible to predict at the initiation of field studies all potential observation or collection opportunities, the number of animals to be encountered, the species to be encountered, or the effects of research procedures.

XXIII.8. The IACUC recognizes that no concise or specific compendium of approved methods for field research encompassing all species, settings, and methods is available, practical, or even desirable.

XXIII.9. The IACUC recognizes that there is considerable variability among taxa of wild vertebrates in terms of their basic needs and how they should be handled, and that the PI is often an authority on the biology of the species under study, and the techniques appropriate for the conduct of the proposed study.

XXIII.10. The IACUC recognizes that the number of specimens required for a field investigation will vary greatly, depending upon the questions being explored, that field studies require larger samples than laboratory studies because field have less control over biotic and abiotic conditions that produce variation than laboratory studies, and that a relatively large number of specimens may actually represent only a very small percentage of the population.

XXIII.11. The IACUC recognizes that state and federal wildlife agencies review applications for permits for their scientific merit and their potential impact on native populations, and issue permits that authorize the taking of specified numbers of individuals, the taxa and methods allowed, the period of study, and often other restrictions designed to minimize the likelihood that an investigation will have deleterious effects.

XXIII.12. The IACUC recognizes that pain perception by many species of vertebrate animals may not be uniform over the various portions of their bodies, and that broad extrapolation of pain perception across taxonomic lines may not be appropriate.

XXIII.13. The IACUC recognizes that the collection of live animals and their preparation as museum specimens is necessary for research and teaching activities in systematic zoology. Each animal collected should serve as a source of information on many levels (e.g., behavior, morphology, genetics), to assure the maximum utility of each animal and to minimize the need for duplicate collecting. Formalin fixation of dead specimens is acceptable; however killing unanesthetized specimens by immersion in a formalin solution is unacceptable.

XXIII.14. IACUC membership includes a biologist who can provide the IACUC with an understanding of the nature and impact of the proposed field investigation, the housing and care of the species to be studied, and the risks associated with maintaining wild vertebrates in captivity, per *IACUC Principles and Procedures III.3*.

XXIII.15. The PI must assure the IACUC in their *wildlife application* that their field study and laboratory use of wild animals will be in accordance with the *Guidelines of the American Society of Mammalogists for the Use of Wild Mammals in Research (*viewable at http://www3.research.usf.edu/cm/docs/ASM-Guidelines_2011.pdf), *Guidelines for the Use of Fishes in Research (*viewable at http://fisheries.org/docs/policy_useoffishes.pdf), *Guidelines for Use of Live Amphibians and Reptiles in Field and Laboratory Research* (viewable at http://www3.research.usf.edu/cm/docs/ASIH_HACC_Amphib_Reptile_Guide.pdf), *Guidelines to the Use of Wild Birds in Research* (viewable at http://www3.research.usf.edu/cm/docs/Wild_Bird_Guidelines_2010.pdf)*, DEA regulations*, *IACUC Principles & Procedures*, *PHS policy*, *AWA*, *Guide*, and AAALAC guidelines.

XXIII.16. The PI assures the IACUC in their *wildlife application* that the taxa chosen is well-suited to answer the research questions posed.

XXIII.17. The PI must make an effort to understand the population status of the taxa to be studied prior to their capture or removal, and ensure that the number of animals used or removed from the wild will be the minimum necessary for accomplishing the goals of the study.

XXIII.18. The PI must ensure that procedures will avoid or minimize distress to the animals consistent with sound research design.

XXIII.19. Procedures that cause more than momentary or slight distress to the animals must be performed with appropriate sedation, analgesia, or anesthesia, except when scientifically justified by the PI in writing and approved by the IACUC.

XXIII.20. Methods of euthanasia must be consistent with the methods recommended by the American Society of Mammalogists, American Society of Ichthyologists and Herpetologists, American Fisheries Society, American Institute of Fisheries Research Biologists, Herpetologists' League, Society for the Study of Amphibians and Reptiles, Ornithological Council, and the *AVMA Guidelines on Euthanasia –* 2013 .

XXIII.21. The PI must have knowledge of all regulations pertaining to the animals under study and have obtained all permits necessary for carrying out the proposed studies prior to their initiation. The PI must ensure that studies conducted outside of the United States will also be in accordance with all wildlife regulations of the country in which the research will be performed.

XXIII.22. Animals of endangered or threatened taxa must not be removed from the wild, nor imported or exported except in compliance with applicable regulations.

XXIII.23. All wild animals are potentially hazardous to research staff, either from traumatic injury, infectious disease, venoms, or poisons. Staff working in the field should maintain current tetanus immunization status, and those working with carnivores or bats should maintain current rabies immunization status. The PI must ensure that the design of the field study does not compromise the health and safety of other animals in the area, or the staff working in the field.

XXIII.24. The PI must assist the University with tracking its field research activities by reporting episodes of wild animal use, the approximate range of taxa, and the approximate number of animals encountered or used to the IACUC c/o Research Integrity & Compliance at intervals appropriate to the study, but at least once each year. Reported animal numbers are tabulated by Comparative Medicine.

XXIII.25. The PI and associated research staff must be familiar with the animals to be studied and their response to disturbance, sensitivity to capture and restraint, and requirements for captive maintenance to the extent that these factors are known or applicable to the study.

XXIII.26. The PI and associated staff must have adequate experience, training, and knowledge regarding the housing, feeding, and care requirements of the animals to be studied, to the extent that these factors are known or applicable to the study, and the PI must direct such activities in the field. The living conditions of animals held at field sites must be appropriate for the involved animals, and contribute to their health and well-being.

XXIII.27. The IACUC acknowledges that although field studies in their simplest form consists of direct observation of free-ranging animals under natural conditions, the objectives of most field studies mandate that individual animals be captured one or more times. Capture techniques that have minimal impact on the animal, and are environmentally benevolent should be used whenever possible. Whenever feasible, the potential for return to the natural environment must be incorporated into the sampling design.

XXIII.28. Acceptable capture techniques that have more than a minimal impact on fish include gill netting, electrofishing, the use of ichthyocides, and the use of hooks or spears; on amphibians and reptiles include trapping and netting; on birds include netting and trapping; and on mammals include trapping, netting, and capture darts which deliver an immobilizing drug. Capture devices such as nets and traps must be checked frequently to prevent animal injuries or mortality.

XXIII.29. Restraint procedures of wild animals, including confinement, physical restrictions, or drug-induced immobilization must be those that cause the least amount of restraint necessary, that can be accomplished in the shortest period of time, that reduce or eliminate contact between the handler and the animal, and that minimize hazards to personnel, whenever possible within the constraints of study design.

XXIII.30. The IACUC acknowledges that the marking of wild animals is a basic method of many field studies, which provides a way of determining the movements, abundance, and population dynamics of wild animals. PIs must carefully consider the nature and duration of restraint required by the marking technique, the amount of tissue affected, whether distress is momentary or prolonged, whether the animal after marking will be at greater than normal risk, whether the animal's desirability as a mate is reduced, and whether the risk of infection or abscess formation is minimal.

XXIII.31. Acceptable marking techniques of fish include fin-clipping, freeze branding, electrocauterization, tagging, radiotelemetry, or radioisotopes; of amphibians and reptiles include scale clipping, banding, tagging, shell marking, radiotelemetry, tattooing, electrocauterization, branding, or radioisotopes; of birds include banding, dyes, collars, tagging, radiotelemetry; of mammals include tagging, banding, radiotelemetry, tattooing, spot-shaving, radioisotopes, or freeze branding. The PI must consider the potential for pain and discomfort associated with each of these techniques, and whether they should be preceded by a general or local anesthetic, and/or followed by a topical antiseptic.

XXIII.32. Maintenance of wild animals in their natural setting must incorporate, as far as possible, those aspects of the natural habitat deemed important to the survival and well-being of the animals. Adequacy of maintenance must be judged by monitoring factors such as appearance, activity level, general behavior, rate of growth, change in body weight, breeding success, and rate of survival. Nutritionally balanced diets must be provided, or natural foods should be duplicated as closely as possible. Natural light, ventilation, temperature, and humidity conditions should be provided, unless these are factors under investigation.

XXIII.33. Methods used for sampling tissues or specimens from wild animals should be designed to obtain the maximal amount of scientific data, with the least amount of animal handling, restraint, and distress, involving a minimum number of animals. Methods that cause more than slight or momentary pain or discomfort require the use of appropriate anesthetics and/or analgesics. Aseptic sampling techniques and surgical procedures must be utilized. PIs must consider whether antimicrobial drugs should be administered following sampling or surgical procedures. The applicant PI is referred to *IACUC Principles and Procedures XV* regarding appropriate aseptic surgical techniques.

XXIII.34. Whenever wild-caught animals are brought into a laboratory, they must be maintained under conditions that comply with the *Guide*, unless the purpose of the study requires the simulation of the natural setting, or when the wild animals housed in the laboratory require conditions other than those prescribed by the *Guide.* In such instances, the design of enclosures and methods of care must accommodate salient features of the animal's ecology, morphology, physiology, and behavior. PIs should consider whether newly captured animals that are brought to the laboratory be quarantined from resident animals for a period of at least 30 days.

XXIII.35. Whenever practical and ecologically appropriate, as soon as possible after capture, upon completion of the study wild-caught animals should be released at the site of the original capture, if their ability to survive has not been impaired, if

they can be expected to function normally, when conditions are conducive to their survival, and when their release is not likely to spread pathogens, unless laws or regulations prohibit release, or release may be detrimental to the well being of the existing native animals.

XXIII.36. All live vertebrate animal activities conducted by University faculty, students, or staff, or supported by University funds, must be proposed to, and approved by the IACUC prior to the initiation of that activity, regardless of where it will be performed.

XXIII.37. Activities involving the collection, preservation, and transportation of specimens from animals that are conducted wholly by non-University personnel, off campus, and not supported by University funds are not regulated by the IACUC.

XXIII.38. The shipping and receiving of biologics, animals or plant specimens must be conducted in accordance with federal safety and importation guidelines and regulations. The PI must act in accordance with the United States Department of Agriculture, Animal and Plant Health Inspection Service regulations regarding the limits on importation of animals or biologics that may have been exposed to an exotic livestock or poultry disease agent, and the limits on the importation of plants and other vegetable matter. The PI must act in accordance with the *Public Health Service Foreign Quarantine Regulations* (42 CFR 71.54) which govern the importation and transfer of etiologic agents and vectors of human disease. The movement of other non-infectious materials such as formalin-fixed tissues, sterile cell cultures, and other preserved tissues or materials where no evidence or indication exists that they contain an infectious agent of animal or public health significance are not governed by these regulations.

XXIV. Conflict of Interest

XXIV.1. No member of the IACUC may participate in the review, discussion, or vote of a project or activity in which he/she has a conflicting interest. The IACUC member will be requested to leave the room prior to the discussion, review, and vote of the project and cannot be counted toward the quorum. The excused member can be recalled to provide information that is requested by the IACUC.

XXIV.2. The IACUC Chairperson will ask IACUC members at the beginning of each meeting if any have a conflicting interest in the business to be conducted that day. In addition, the IACUC administrative support staff of Research Integrity & Compliance will provide the IACUC Chairperson with a list of IACUC members who are identified as participants in projects/activities being reviewed or discussed during the convened meeting. The IACUC Chairperson will ask all members with conflicting interests to leave at the appropriate time during the proceedings.

XXV. Public Records

XXV.1. University research records are subject to both federal and state laws. Requests for information under the federal Freedom of Information Act (FOIA) should be made to Research Integrity & Compliance at 974-3234. Requests for information under the Florida Public Records Law should be made to USF General Counsel at 974-2734.
